# Cultural validation and language translation of the scientific SCI exercise guidelines for use in Indonesia, Japan, Korea, and Thailand

**DOI:** 10.1080/10790268.2021.1945857

**Published:** 2021-07-06

**Authors:** Yukio Mikami, Damayanti Tinduh, KunHo Lee, Chayaporn Chotiyarnwong, Jan W. van der Scheer, Kyung Su Jung, Hiroshi Shinohara, Inggar Narasinta, Seung Hyun Yoon, Napatpaphan Kanjanapanang, Takafumi Sakai, Martha K. Kusumawardhani, Jinho Park, Pannika Prachgosin, Futoshi Obata, Ditaruni Asrina Utami, Phairin Laohasinnarong, Indrayuni Lukitra Wardhani, Siraprapa Limprasert, Fumihiro Tajima, Victoria L. Goosey-Tolfrey, Kathleen A. Martin Ginis

**Affiliations:** 1 Department of Rehabilitation Medicine, Wakayama Medical University, Wakayama, Japan; 2 Department of Physical Medicine and Rehabilitation, Faculty of Medicine, Universitas Airlangga Dr. Soetomo General Academic Hospital, Surabaya, Indonesia; 3 Department of Prescription and Rehabilitation of Exercise, Dankook University, Cheonan, Republic of Korea; 4 Department of Rehabilitation Medicine, Faculty of Medicine Siriraj Hospital, Mahidol University, Bangkok, Thailand; 5 THIS Institute, Department of Public Health and Primary Care, University of Cambridge, Cambridge, UK; 6 Peter Harrison Centre for Disability Sport, School of Sport, Exercise & Health Sciences, Loughborough University, Loughborough, UK; 7 Medical Center for Health Promotion and Sport Science, Wakayama Medical University, Wakayama, Japan; 8 Faculty of Health Sciences, Aomori University of Health and Welfare, Aomori, Japan; 9 Department of Physical Medicine and Rehabilitation, Ajou University School of Medicine, Suwon, Republic of Korea; 10 Department of Physical Therapy, Takarazuka University of Medical and Health Care, Takarazuka, Japan; 11 Department of Counseling, Health and Kinesiology, College of Education and Human Development, Texas A&M University-San Antonio, San Antonio, Texas, USA; 12 Department of Medicine, Division of Physical Medicine & Rehabilitation, University of British Columbia, Vancouver, Canada; 13 School of Health and Exercise Sciences, University of British Columbia, Kelowna, Canada; 14 International Collaboration on Repair Discoveries (ICORD), University of British Columbia, Vancouver, Canada; 15 Centre for Chronic Disease Prevention and Management, Southern Medical Program, University of British Columbia, Kelowna, Canada

**Keywords:** Tetraplegia, Paraplegia, Physical activity, Rehabilitation, Health promotion, Fitness, Cardiometabolic health

## Abstract

**Context:**

Indonesia, Japan, Korea, Thailand.

**Objective:**

To culturally validate and translate the Scientific Exercise Guidelines for Adults with Spinal Cord Injury (SEG-SCI) for use in four Asian countries.

**Design:**

Systematic Review

**Participants:**

N/A

**Methods:**

A systematic review was conducted to identify all published English- and local-language studies conducted in Indonesia, Japan, Korea, and Thailand, testing the effects of exercise training interventions on fitness and cardiometabolic health in adults with acute or chronic SCI. Protocols and results from high-quality controlled studies were compared with the SEG-SCI. Forward and backward translation processes were used to translate the guidelines into Bahasa Indonesian, Japanese, Korean and Thai languages.

**Results:**

Fifteen studies met the review criteria. At least one study from each country implemented exercise prescriptions that met or exceeded the SEG-SCI. Two were controlled studies. In those two studies, relative to control conditions, participants in exercise conditions achieved significant improvements in fitness or cardiometabolic health outcomes only when the exercise intervention protocol met or exceeded the SEG-SCI. During the language translation processes, end-users confirmed that SEG-SCI language and terminology were clear.

**Conclusion:**

Clinical researchers in Indonesia, Japan, Korea and Thailand have implemented exercise protocols that meet or exceed the SCI-SEG. Results of high-quality studies align with the SEG-SCI recommendations. Based on this evidence, we recommend that the SEG-SCI be adopted in these countries. The cultural validation and translation of the SEG-SCI is an important step towards establishing consistent SCI exercise prescriptions in research, clinical and community settings around the world.

## Introduction

The Scientific Exercise Guidelines for Adults with Spinal Cord Injury (SEG-SCI) stipulate the minimum dose of exercise required to achieve significant improvements in fitness and in cardiometabolic health.^1,2^ The fitness guideline stipulates that for cardiorespiratory fitness (i.e. the ability of the circulatory and respiratory systems to supply fuel during sustained physical activity^3^) and muscle strength benefits, adults with spinal cord injury (SCI) should engage in at least 20 min of moderate to vigorous intensity aerobic exercise and strength-training exercises twice per week. The cardiometabolic health^4^ guideline stipulates that for improvements in risk factors associated with the development of type 2 diabetes mellitus and cardiovascular disease, adults with SCI are suggested to engage in at least 30 min of moderate to vigorous intensity aerobic exercise three times per week.

An international group of scientists, clinicians, people with SCI and other stakeholders comprised the guideline development team. The SEG-SCI^1^ were formulated using the same systematic, evidence-based approach that has been used by the World Health Organization and other national organizations to develop physical activity guidelines for the general population.^5^ Development of the SEG-SCI^1^ was motivated by the need for consistent, evidence-based exercise prescriptions for people with SCI. Publication of the SEG-SCI^1^ is a significant milestone for advancing the scientific and clinical application of exercise to improve the health and well-being of people living with SCI, worldwide.

During the guideline development process, three expert panels were convened with representatives from Australia, Canada, Europe, the United Kingdom, and the United States. The SEG-SCI^1^ were formulated after extensive deliberation of an evidence base consisting of over 200 English-language studies. These studies tested the effects of exercise interventions on fitness, cardiometabolic health and bone health among adults with acute and chronic SCI.^6^ A limitation of this process was that many geographic regions - such as Asia, Africa, South America - were not represented on the guideline panel and relatively few studies from these regions were included in the evidence base, perhaps because the search was limited to English-language publications. Guidelines should be relevant to the context and the people who will use them.^7^ It is unlikely that a guideline will be adopted if it conflicts with local research and practice. Furthermore, adoption of a guideline dramatically increases if the people who will use the guideline have a stake, or involvement, in its production.^8^ Therefore, before recommending and investing in the implementation of the SEG-SCI^1^ in other regions, it is important to consider whether the local research evidence supports or refutes the guidelines^9^ and to involve local stakeholders in the cultural validation and translation of the SEG-SCI.

This paper reports on systematic processes to culturally validate and translate the SEG-SCI^1^ for use in four Asian countries: Indonesia, Japan, Korea, and Thailand. These four countries were brought together for this project for several reasons. First, Japan, Korea and Indonesia have hosted the Asian Para Games and Thailand hosted the precursor FESPIC Games (Far East and South Pacific Games for the Disabled). Because of their involvement in disability sport, these countries are eager to develop initiatives to advance health and physical activity in their countries. Second, the four countries have different languages, culture, history and religion. Japan and Korea are located in East Asia while Indonesia and Thailand are located in South Asia. Altogether, the four countries provide a good representation of the diversity of democratic countries in Asia. Third, scientists from these countries had a pre-established memorandum of understanding with the project funding partner (Wakayama Medical University), which helped to facilitate the project and flow of resources.

Our first project objective was to culturally validate the SEG-SCI^1^ through a systematic review of all published (English and local language) research conducted in Indonesia, Japan, Korea, and Thailand; specifically, research that has assessed the effects of exercise interventions on fitness and cardiometabolic health in adults with SCI. Because the SEG-SCI are based on the highest quality evidence available,^1, 6^ we were primarily interested in the results of the highest quality studies (i.e. randomized, controlled studies) from these countries. Similar to the process by which theories are validated by testing their predictions against observations, we tested the cultural validity of the guidelines by testing the following predictions: (1) exercise training studies that implemented exercise protocols that met or exceeded the SEG-SCI^1^ would report significant fitness and cardiometabolic health outcomes in the exercise condition relative to the control condition; and (2) exercise training studies that implemented exercise protocols that did not meet or exceed the SEG-SCI^1^ would not report significant fitness and cardiometabolic health outcomes relative to the control condition.

Our second objective was to translate the SEG-SCI^1^ into Bahasa Indonesian, Japanese, Korean and Thai languages. This process involved forward and backward translations of the guidelines and engagement with end-users (e.g. medical doctors/physiatrists, SCI patients, physiotherapists, occupational therapists) to ensure that the guideline language and terminology were clear and appropriate to the cultural context and the users of the guidelines.

## Method

### Systematic review

The purpose of the systematic review was to identify all relevant studies conducted in Indonesia, Japan, Korea and Thailand that fit within the parameters of the review used to formulate the SCI-SEG.^1, 6^ The question addressed in the systematic review was: What are the effects of exercise training interventions on cardiorespiratory fitness, muscle strength and cardiometabolic health outcomes, in adults with SCI?

Cardiorespiratory fitness refers to the capacity of the respiratory and circulatory systems to transport oxygen from the atmosphere to skeletal muscle mitochondria to perform physical activity.^3, 10^ In SCI research settings, cardiorespiratory fitness is typically measured as the maximum volume of oxygen consumed from the peak work rate achieved on an arm ergometer.^6^ Muscular strength refers to the amount of external force that a muscle can exert.^3^ In SCI research, muscular strength is typically measured as the maximum amount of weight a person can lift with a particular muscle group, or the maximum amount of force that can be exerted by a particular muscle group.^6^ Cardiometabolic disease refers to a spectrum of health conditions that begin with insulin resistance, progress to metabolic syndrome (characterized by high blood pressure, high fasting blood sugar, high triglycerides, low HDL cholesterol, and obesity), pre-diabetes, and finally to more severe conditions including type 2 diabetes mellitus and cardiovascular disease.^4^ Examples of cardiometabolic health and cardiometabolic disease indicators used in SCI research include fasting glucose, glucose tolerance, waist circumference, serum HDL cholesterol, blood pressure and fasting triglycerides.^6^

Studies were obtained from several sources and searches. First, the original review^6^ was used to identify studies conducted in the four Asian countries, and published in English between January 1, 1980 and January 16, 2016. Second, the English-language search from the original review^6^ was updated in PubMed, MEDLINE, PsychINFO, SPORTDiscus, EMBASE, and CINAHL to identify relevant studies conducted in the four countries, and published in English between January 1, 2016 and December 30, 2019. Third, in each country, local databases were searched to identify relevant studies conducted in that country and published in the local language between January 1, 1980 and December 31, 2019. The databases searched in each country were:

- Indonesia: Google Cendekia scholar.google.co.id and Garuda garuda.ristekdikti.go.id;
- Japan: Japan Medical Abstracts Society and CiNii
- Korea: Research Information Service System (RISS), Korean studies Information Service System (KISS), and DataBase Periodical Information Academic (DBpia);
- Thailand: Thai-Journal Citation Index (TCI).

All search strategies combined local language search terms that represent SCI (e.g. “paraplegia,” “tetraplegia”) and exercise interventions (e.g. “exercise,” “strength training”). The search terms and databases used for each search are presented in Supplementary File 1. The review protocol (not registered) and reporting methods followed the Preferred Reporting Items for Systematic Reviews and Meta-Analyses (PRISMA).^11^ Studies were included that:

- had a sample with at least 50% adults (≥16 years) with traumatic or non-traumatic SCI, excluding multiple sclerosis and spina bifida;
- implemented an exercise intervention (defined as exercise training sessions occurring for ≥2 weeks), employing any type of exercise, but with at least one of the following parameters reported: type (e.g. upper-body aerobic exercise, ambulation exercise), frequency (e.g. number of sessions per week), intensity (e.g. % peak oxygen uptake) or duration (e.g. minutes per session);
- used any study design except for case studies, which were excluded because of their relatively strong potential for bias. Studies were considered controlled if the control group did not receive an exercise intervention. If the control/comparator group received an exercise intervention, the study was considered a pre–post design and only the within-groups data were included. Usual care was accepted as a control condition if the exercise condition also received usual care in addition to exercise;
- reported at least one measure of any of the following outcomes: cardiorespiratory fitness, power output, muscle strength, body composition, cardiovascular risk factors, or bone health.^6^

Data were extracted from eligible studies and tabulated using the same tables employed in the original review.^6^ For eligible studies that were included in the original review, previously extracted data^6^ were copied into a new table for this project, and verified by author KMG. For eligible studies identified through the new searches, data were extracted by the author who spoke the language of publication, compiled into a table, and verified by a second reader. Authors KMG and KSJ then compiled the individual tables for each review into a single table (Supplementary File 2).

To assess the quality of each study, risk of bias was assessed using a 10-item version of the Physiotherapy Evidence Database (PEDro tool) for randomized controlled trials and a modified Downs and Black scale for all other study designs.^12^ Because it is nearly impossible to blind participants in an exercise RCT, the Downs and Black scale was modified to credit all studies for the item “blinding of all subjects”. Using a four-level rating system developed by Spinal Cord Injury Research Evidence (SCIRE),^12^ studies were then classified as Level 1 (RCTs with a PEDro score ≥ 6), Level 2 (e.g. RCTs with a PEDro score ≤ 6), Level 3 (e.g. uncontrolled pre–post studies with a Downs and Black score ≥ 21) or Level 4 (e.g. uncontrolled pre–post studies with Downs and Black score < 21). The evidence was rated in this manner because we wanted to use evidence from only the highest quality studies (i.e. Level 1 and 2 studies) to test the predictions for validating the guidelines.

### Language translation

The English-language SEG-SCI document^1^ was forward translated into Bahasa Indonesian, Japanese, Korean and Thai languages. The Indonesian translation was completed by an English teacher and the other three translations were completed by physiatrists. Each translation was reviewed by a convenience sample of end-users who provided feedback on the clarity of language and terminology. At least two rounds of feedback were sought for each translation. The guidelines were then back-translated to English by another physiatrist (Indonesian, Thai) or a translator (Japanese, Korean).

## Results

A total of 15 studies met the review inclusion criteria. Information about these studies and their references are presented in Supplementary File 2. Five of 15 studies were English-language studies included in the original review (one from Japan and four from Korea). Four additional English-language studies were identified through the updated search (three from Japan and one from Korea). Searches of the local-language literature yielded one study from Indonesia, two from Japan, one from Korea and two from Thailand that met the review criteria. PRISMA flow diagrams for the five new searches are presented in Supplementary File 1.

Ten studies focused on people with chronic SCI (>12 months post-injury), two studies included people with acute SCI (≤ 12 months post-injury), and three studies did not report time since injury. In all studies where the exercise location was reported, the exercises were performed in a supervised clinical setting. Regarding the quality of the studies, the majority (12/15) were Level 4. One study each was classified as Level 1, Level 2, and Level 3. We focus the validation process on testing our predictions using the evidence derived from the highest quality studies. Therefore, the following sections discuss only the results of the Level 1^13^ and 2^14^ RCTs. Details of these two studies are presented in Table 1.

**Table 1:** Characteristics of the Level 1 and Level 2 Studies Used to Validate the Scientific Exercise Guidelines for Adults with Spinal Cord Injury.

Study and Level of Evidence	Kim et al. 2015^14^ (Level 2 RCT)			Kim et al. 2019^13^ (Level 1 RCT)		
Participants	Chronic SCI N=15; M9/F6 Mean Age: 33 ± 6 (22-46) y Mean Time Since Injury: 7 ± 4 (2-16) y Injury Characteristics: AIS A-B; C5-T11			Chronic SCI N=19; M12/F7 Mean Age: 36.8 ± 6.9 (23-53) y Mean Time Since Injury: 9.23 (2-27) y Injury Characteristics: AIS A,B,C; C4-L1		
Intervention + Control Conditions	Exercise: Stationary hand cycling using wheelchair with add-on arm crank and game-like software Control: No exercise intervention			Exercise: Resistance, circuit, and arm ergometry or hand-cycling Control: Standard Care		
Relative intensity	Progressive over weeks: 70–80% HRpeak or 5–7 on Borg CR10 scale			Progressive over weeks 65-85% max HR on Borg CR10 scale		
Duration (min)	36 min (12 sets of 3-min exercise interspersed with 1-min rest) total of 60 min incl. warm-up and cool-down			60 min (25 min warm-up) [5 min joint exercises, 15 min arm ergometer, 5 min stretching], resistance training [1-3 sets, 10–20 reps], circuit training [1-2 sets], aerobic training [10-20 min]		
Frequency (times/week)	3			3		
Intervention period (weeks)	6			6		
Outcome Measures		Pre M ± SD	Post M ± SD		Pre M ± SD	Post M ± SD
	
**1. Cardiorespiratory Fitness**						
VO2 peak (ml/kg/min)	Exercise:	16.8 ± 7.2	21.2 ± 9.1*^#^	Exercise:	11.7 ± 8.1	15.2 ± 9.6*
	Control:	15.2 ± 3.4	13.7 ± 3.4*	Control:	9.4 ± 4.5	11.7 ± 7.9
**2. Muscle Strength**						
Elbow flexion (N)	Exercise:	123.3 ± 58.9	177.1 ± 73.2*^#^	Exercise:	169.0 ± 73.0	231.0 ± 96.1*^#^
	Control:	152.8 ± 35.1	148.7 ± 35.0*	Control:	202.8 ± 64.4	199.5 ± 68.5
Elbow extension (N)	Exercise:	34.0 ± 46.0	44.4 ± 59.8*^#^	Exercise:	95.7 ± 81.9	128.6 ± 101.3*^#^
	Control:	18.0 ± 18.3	17.2 ± 17.5	Control:	94.5 ± 82.1	94.5 ± 87.9
Shoulder abduction (N)	Exercise:	96.8 ± 57.7	134.6 ± 65.7*^#^	Exercise:	118.7 ± 61.5	159.0 ± 71.3*^#^
	Control:	106.4 ± 27.2	100.9 ± 25.1	Control:	134.6 ± 60.7	135.0 ± 69.6
Shoulder adduction (N)	Exercise:	104.7 ± 63.5	142.3 ± 82.5*^#^	Exercise:	124.3 ± 51.6	165.1 ± 65.2*^#^
	Control:	92.7 ± 34.7	90.2 ± 37.0	Control:	115.1 ± 69.3	116.3 ± 75.4
Shoulder flexion (N)	Exercise:	111.2 ± 65.6	142.8 ± 74.6*^#^	Exercise:	120.0 ± 55.6	159.6 ± 68.4*^#^
	Control:	105.2 ± 30.5	102.9 ± 29.2	Control:	140.3 ± 67.0	141.1 ± 72.5
Shoulder extension (N)	Exercise:	98.7 ± 63.0	139.6 ± 73.5*^#^	Exercise:	128.6 ± 29.4	172.6 ± 38.8*^#^
	Control:	132.8 ± 29.2	128.7 ± 25.8	Control:	143.1 ± 71.0	144.8 ± 79.9
**3. Body Composition**						
Lean Mass (kg)	Exercise:	20.2 ± 5.0	21.7 ± 4.9	Exercise:	21.9 ± 5.2	22.0 ± 4.9
	Control:	19.0 ± 6.4	19.4 ± 6.3	Control:	19.3 ± 6.6	19.7 ± 7.0
Body Fat (%)	Exercise:	39.0 ± 13.7	35.5 ± 11.8	Exercise:	35.3 ± 10.8	33.5 ± 10.3*
	Control:	40.6 ± 6.8	40.5 ± 6.4	Control:	39.6 ± 5.6	38.4 ± 7.1
BMI (m/kg^2^)	Exercise:	22.0 ± 3.7	21.7 ± 3.5*^#^	Exercise:	21.8 ± 2.9	21.3 ± 2.8*
	Control:	20.8 ± 2.7	21.1 ± 2.7	Control:	20.8 ± 1.9	20.6 ± 2.0
Waist Circumference (cm)	Exercise:	88.3 ± 13.1	85.6 ± 12.2*^#^	Exercise:	84.1 ± 11.9	82.5 ± 11.4^#^
	Control:	81.7 ± 9.0	82.6 ± 8.7	Control:	79.4 ± 6.6	79.2 ± 6.4
**4. Cardiometabolic Risk Factors**						
HOMA-IR	Exercise:	1.0 ± 0.6	0.6 ± 0.3*^#^	Exercise:	1.5 ± 1.0	0.9 ± 0.4*^#^
	Control:	1.1 ± 0.8	1.6 ± 1.3	Control:	0.5 ± 0.2	0.6 ± 0.2
Fasting Insulin (μU/ml)	Exercise:	5.4 ± 2.9	3.4 ± 1.5*^#^	Exercise:	7.5 ± 4.7	4.5 ± 2.2*^#^
	Control:	4.9 ± 2.9	6.7 ± 4.5	Control:	2.9 ± 1.1	3.2 ± 1.3
HDL-C (mg/dl)	Exercise:	42.2 ± 11.5	46.1 ± 12.3*	Exercise:	48.7 ± 21.3	54.3 ± 18.4*^#^
	Control:	45.1 ± 7.0	44.7 ± 5.8	Control:	51.2 ± 10.6	49.4 ± 10.6
LDL-C (mg/dl)	Exercise:	113.1 ± 25.9	110.7 ± 28.8	Exercise:	93.5 ± 31.2	89.0 ± 27.0
	Control:	118.7 ± 23.9	117.7 ± 25.0	Control:	125.6 ± 29.3	139.6 ± 20.3
TC (mg/dl)	Exercise:	176.2 ± 35.7	177.2 ± 35.8	Exercise:	162.3 ± 34.1	169.0 ± 25.5
	Control:	183.1 ± 21.4	179.5 ± 29.6	Control:	199.5 ± 34.0	213.8 ± 22.7
Triglycerides (mg/dl)	Exercise:	103.0 ± 42.0	102.1 ± 29.3			
	Control:	96.4 ± 49.2	85.4 ± 22.3			
Glucose (mg/dl)	Exercise:	78.6 ± 8.3	77.0 ± 7.7	Exercise:	81.0 ± 5.4	79.3 ± 5.4
	Control:	88.7 ± 10.8	89.4 ± 12.8	Control:	75.6 ± 3.6	7.5 ± 3.0

Note. * indicates a significant within-group, pre-post improvement, but no difference compared to the control group;

# indicates a significant improvement compared to the control group;

*# indicates a significant pre-post improvement and a significant improvement compared to the control group

The Level 1 RCT^13^ was conducted in Korea and published in English in 2019. Participants were 12 men and 7 women with chronic SCI, lesion levels C4 to L1, and AIS levels A-C. The control condition received standard care. In the exercise condition, participants completed a supervised, 60-minute combined exercise program, 3 times per week, for 6 weeks. The program was progressive and consisted of a 25-minute warm-up, resistance training (1-3 sets of each exercise), circuit training (1-2 sets), and 10–20 min of arm ergometry or hand-cycling performed at 65%-85% of maximum heart rate. Relative to the control condition, participants in the exercise condition significantly increased their strength, but not their cardiorespiratory fitness (measured as V̇O_2peak_).

These results can be interpreted relative to the SEG-SCI.^1^ When compared to the SEG-SCI^1^ to improve fitness, and expressed as weekly volumes of exercise (see Table 2), the RCT’s strength-training exercise prescription (6-15 sets per week) met the guidelines recommendation for strength-training (minimum 6 sets per week). The RCT finding of significant increases in strength, relative to a control condition, is interpreted as support for the guideline. Conversely, the minimum amount of aerobic exercise prescribed in the RCT (30 min per week) was less than the SEG-SCI’s^1^ recommendation for aerobic exercise (minimum 40 min per week; see Table 2). Thus, aerobic exercise performed *below* the SEG-SCI^1^ recommendation did *not* significantly improve cardiorespiratory fitness. Participants in the exercise condition also showed significant improvements in waist circumference, high-density lipoprotein cholesterol (HDL-C), insulin resistance, and fasting insulin compared to the control condition. The authors noted, however, that they could not differentiate whether reductions in insulin resistance and fasting insulin were a carryover effect from the last bout of exercise (which can last for 48–72 hours^15^) or were a chronic effect of the exercise training.

**Table 2:** Comparison between the SCI Scientific Exercise Guidelines and the Exercise Prescriptions Used in the Level 1 and 2 Studies.

SCI Scientific Exercise Guidelines			Level 1 + 2 Studies			
Guideline		Weekly Equivalent	Exercise Prescription	Weekly Equivalent	Meets the Guidelines?	Results
			Level 1^13^			
Fitness	At least 20 min of moderate to vigorous intensity aerobic exercise 2 times/week.	At least 40 min.	10-20 min of arm ergometry or handcycling at 65-85% of HR mas, 3 times/week.	30-60 min.	No. (Minimum is less than guideline minimum.)	No significant increases in cardiorespiratory fitness relative to a control group.
	At least 3 sets of strength-training exercises for each major functioning muscle group at a moderate to vigorous intensity, 2 times/week.	At least 6 sets.	Resistance training (1-3 sets of each exercise) + circuit training (1-2 sets), 3 times/week.	6–15 sets	Yes.	Significant increases in strength relative to a control group.
Cardio-metabolic Health	At least 30 min of moderate to vigorous intensity aerobic exercise 3 times/week.	At least 90 min.	Level 2^14^			
			36 min of handcycling at 70-80% HR peak, 3 times/week.	108 min.	Yes.	Significant increases in cardiorespiratory fitness; significant decreases in BMI, waist circumference, insulin, and fasting insulin relative to control group.

The Level 2 RCT^14^ was conducted in Korea, and published in English in 2015. Participants were 9 men and 6 women with chronic SCI, lesion levels C5 to T11 and AIS levels A-B. Participants randomized to the exercise condition performed 36 min of supervised handcycling at 70-80% of their peak heart rate, 3 times per week, for 6 weeks. Compared to the control condition, participants in the exercise condition had significant increases in cardiorespiratory fitness (measured as V̇O_2peak_) and significant decreases in body mass index, waist circumference, insulin resistance and fasting insulin. Expressed as a weekly volume of exercise, the RCT exercise prescription (108 min per week) met and exceeded the SCI cardiometabolic health exercise guideline (90 min per week). Overall, the findings of significant improvements in cardiometabolic health indicators are interpreted as support for the SEG-SCI^1^ with the caveat that insulin was measured within two days of the last exercise session.

There were no Level 1 or 2 studies from Indonesia, Japan, or Thailand. The Level 4 studies from these countries were not used as evidence for testing the predictions regarding the effectiveness of the guidelines. However, in the interest of evaluating the cultural appropriateness of the guidelines, it should be noted that from each country, there was at least one study that implemented exercise prescriptions that met or exceeded the SEG-SCI.^1^ For instance, an Indonesian pre–post study reported that arm ergometry performed for 25 min at 70-85% of maximum heart rate, 3 times per week, significantly improved cardiorespiratory fitness in men and women with chronic SCI.^16^ Similarly, a Japanese pre–post study reported that 60 min of arm ergometry performed at 50-70% of heart rate reserve, 4 times per week, significantly increased cardiorespiratory fitness (measured as V̇O_2peak_), and decreased body mass, waist circumference, systolic blood pressure, triglycerides and plasminogen activator-inhibitor-1 (PAI-1) in men with chronic SCI.^17^ A Thai pre–post study reported that strengthening exercises performed 5 times per week significantly increased abdominal and back muscle strength in men and women with SCI.^18^ While we are reluctant to draw conclusions from these Level 4 studies regarding the effectiveness of the guidelines, the studies indicate that researchers and clinicians in these Asian countries have delivered exercise prescriptions that meet or exceed the SCI-SEG.^1^

Only four studies mentioned adverse events. No serious adverse events were reported.

### Translation

Each forward translation was reviewed by at least 10 end-users (median = 12; range = 10-17; see Table 3). Minor adjustments to language were made after each round of review to ensure a clear and appropriate description of the exercise type, frequency, intensity and duration specified in the SEG-SCI.^1^ During this process, end-users in each country raised questions about *how* to do the exercise (e.g. what specific strength-training exercises should be performed; how to monitor and assess exercise intensity). Information on *how* to exercise is beyond the scope of the SEG-SCI.^1^ We elaborate on this issue in the Discussion.

**Table 3.** Characteristics of Translators and Reviewers Engaged During the Translation Process in Each Country.

	Indonesia	Japan	Korea	Thailand
Translator Occupation	English teacher	Physiatrist	Physiatrist	Physiatrist
**1^st^ Reviewers Total number**	**9**	**4**	**6**	**6**
MD Physiatrists	3	4	2	2
Physiotherapists	3		3	2
Occupational therapists			1	2
Secretary/administration staff	3			
**2nd Reviewers Total number**	**8**	**4**	**5**	**4**
MD including Physiatrists	2	2	1	2
Physiotherapists		1	1	
Occupational therapists				
Secretary/administration staff		1		
patients	6		3	2
**3rd Reviewers Total number**	** **	**5**	** **	** **
patients		5		
Back-Translator Occupation	Physiatrist	Translator	Translator	Physiatrist

After the back translation, some minor changes in wording were applied to the translations to ensure consistency with the original English version (see Supplementary File 4 for a description of these changes). For example, in Bahasa Indonesian, the word cardiorespiratory directly translates to “pernafasan” or “breathing”. A direct translation of the SEG-SCI^1^ could therefore mislead Indonesian end-users to believe these are exercises to improve their breathing. Working with a physiatrist on the translation, we arrived at the word “Jantung-paru” which translates to “heart-lung”, and is more indicative of cardiopulmonary fitness. This example speaks to the importance of a collaborative translation approach to ensure that culturally appropriate language and terminology are used; otherwise, the intent and uptake of the guidelines could be severely compromised.

**Figure 1:**
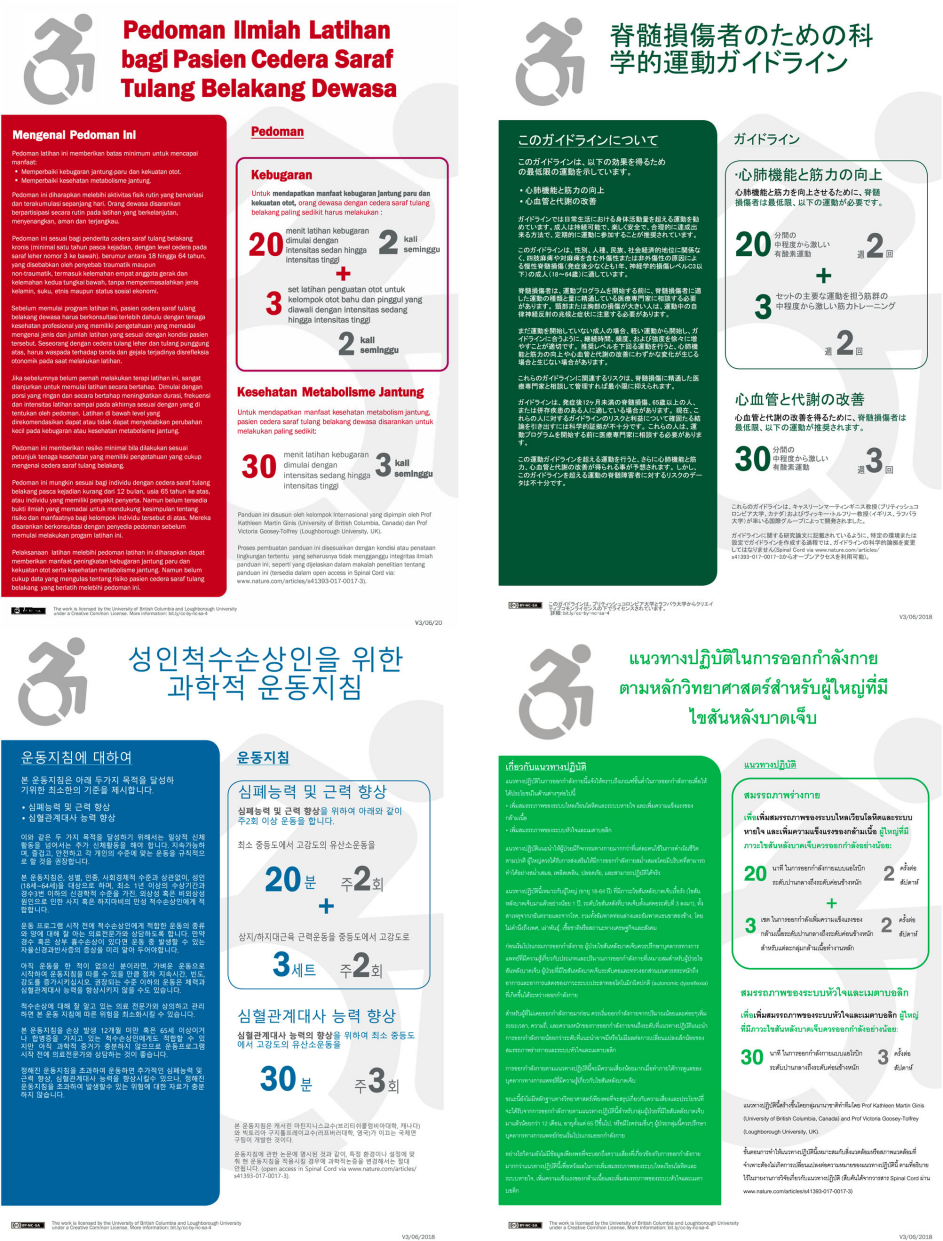
Indonesian, Japanese, Korean and Thai language translations of the Scientific SCI Exercise Guidelines. Available from https://sciactioncanada.ok.ubc.ca/sci-action-international/ and https://www.ncsem-em.org.uk/sciguidelinesasia/

The final versions of the Bahasa Indonesian, Japanese, Korean and Thai-language translations of the SEG-SCI^1^ are presented in Figure 1.

## Discussion

The overarching purpose of this project was to culturally validate and translate the SEG-SCI^1^ for use in Indonesia, Japan, Korea and Thailand. The first step in this process involved a systematic review of relevant, local research. The results from a Level 1 and a Level 2 RCT, both conducted in Korea, supported our prediction that improvements in fitness and cardiometabolic health outcomes would only occur with exercise interventions that met or exceeded the SEG-SCI.^1^ The relatively low proportion of Level 1 and 2 studies (13.3%) was similar to the proportion identified in the review underpinning the SEG-SCI^1^ (16.6%)^6^ and is a testament to the myriad challenges associated with conducting controlled exercise interventions among people with SCI (e.g. participant recruitment and retention^19^). While there were no Level 1 or 2 studies from Indonesia, Japan, or Thailand, each of these countries had a Level 4 study that implemented exercise that met or exceeded the SEG-SCI,^1^ which suggests that the types and amounts of exercise recommended in the SEG-SCI^1^ are appropriate in these countries. Taking this evidence together, we recommend that the SEG-SCI^1^ be adopted in Indonesia, Japan, Korea and Thailand.

### Research issues identified through the systematic review

The systematic review brought several noteworthy issues to light. First, the exercise prescriptions used in the studies varied tremendously in terms of exercise frequency, intensity, type and duration. Inconsistency in exercise prescriptions is a common limitation of the SCI exercise literature,^6^ along with a lack of detailed information on how people were trained to do the exercises and the level of supervision they received. These inconsistencies make it difficult to compare findings across studies and to identify the precise dose of exercise that will lead to a specific outcome. For instance, the SEG-SCI^1^ specify the dose of exercise required to improve cardiometabolic health as a *general* outcome. However, the precise dose of exercise required to elicit clinically significant improvements in *specific* cardiometabolic health outcomes (e.g. blood lipids) for people with SCI it is not yet known. The SEG-SCI^1^ should be used as a standardized exercise prescription in future RCTs. Scientists can then test the effects of this prescription on specified outcomes, including outcomes that are not currently addressed by the SEG-SCI^1^ due to a lack of evidence (e.g. bone health, psychological well-being, pain^20^). Scientists can also compare the effects of other exercise prescriptions (e.g. a prescription based on a high intensity interval training protocol^21^) relative to the effects of exercise performed according to the SEG-SCI.^1^

A second observation was the wide range of outcome measures used to assess each of the six outcome categories (e.g. cardiovascular risk factors, muscle strength). Scientists would benefit from developing a common set of outcome measures for use in SCI exercise intervention studies. Third, consistent with trends observed in the SEG-SCI^1^ evidence base,^6^ very few studies included people with acute SCI. Given the lack of high-quality studies involving adults with acute SCI, it was not possible for the guideline panel to formulate an exercise guideline recommendation for this sub-group.^1^ Likewise, based on the existing scientific evidence, we are not able to confirm the validity of the SEG-SCI^1^ when used by people with acute SCI in Indonesia, Japan, Korea or Thailand. Fourth, none of the studies compared outcomes across participants with different injury characteristics (e.g. lesion levels). This trend was also observed in the SEG-SCI^1^ evidence base^6^ whereby sample sizes were generally too small to conduct meaningful sub-analyses. Another observation was that, consistent with the studies in the SEG-SCI^1^ evidence base,^6^ all of the exercise interventions were conducted in supervised rehabilitation settings. Given the limited supervised exercise settings available to people with SCI, and the countless environmental barriers to exercise experienced by people with SCI, it will be important to test the effectiveness of the SCI-SEG^1^ when exercise is performed in unsupervised home and community settings.^20^ Finally, adverse events were rarely documented in the reviewed studies. Going forward, it is important for researchers to track and report adverse events in order to provide further assessment of the risks and benefits associated with exercise interventions.

### Dissemination and implementation of the translated guidelines

An important outcome of this project is the production of Bahasa Indonesian, Japanese, Korean and Thai language versions of the SEG-SCI.^1^ By conducting the translation process in collaboration with end-users of the guidelines, we can be reassured that the SCI-SEG^1^ have retained their intent, detail, and scientific accuracy while being consistent with the terminology and language used by each culture to describe exercise and its benefits. These translations can now be disseminated to educate people with SCI and health-care providers regarding *what* people with SCI should do to improve their fitness and cardiometabolic health. Additional supporting resources (e.g. pamphlets, videos), tailored to local audiences and settings, will be required to educate and motivate end-users on *how* to achieve the guidelines in community and clinical settings.^1,9^ During the translation process, people with SCI and health-care providers raised questions about *how* to do the SEG-SCI^1^ (e.g. what specific strength-training exercises to do; how to monitor their exercise intensity). These questions were not surprising given the purpose of the SEG-SCI^1^ is to stipulate *what* type of exercise and *what* minimum frequency, duration and intensity of exercise are needed to elicit significant fitness and cardiometabolic health outcomes. Scientific exercise guidelines do not specify *how* to exercise. To be maximally effective, exercise guidelines should be presented along with evidence-informed resources that educate and motivate people to achieve the guidelines.^22^

A 10-step knowledge-translation process has been formulated to guide the development of resources to support implementation of the SEG-SCI^1^ in community and clinical practice settings.^23^ Key to this process, is an understanding of the needs and preferences of people who will use the SEG-SCI^1^ (e.g. people living with SCI, rehabilitation specialists) and their barriers to implementing and adhering to the guidelines. Although factors associated with exercise adherence in people with SCI have been studied extensively in European and English-speaking countries,^24,25^ there is very little published research on this topic from Asian countries.

In addition to barriers frequently reported in other regions, such as a lack of accessible transportation, exercise facilities, and equipment,^24,25^ there may be unique barriers to physical activity participation in Asian countries. For instance, Indonesia is an archipelago of over 17,000 islands, 6,000 of which are inhabited and many are remote.^26^ It is virtually impossible for the limited number of local SCI health and rehabilitation specialists^27^ to support the exercise needs of all Indonesians living with SCI. Similarly, Thailand faces challenges of insufficient numbers of SCI rehabilitation professionals working in rural and remote areas of the country.^28^ Tele-rehabilitation and internet-delivered physical activity interventions can help overcome access barriers in some countries, but Indonesia and Thailand lag below the global average for digital connectivity.^29^ E-health solutions will have limited reach in these countries.

In Japan, a unique barrier to SCI exercise guideline uptake and adherence is that Japan does not have the same exercise and fitness culture as western societies such as Canada, the United States and many European countries.^30^ Epidemiological data indicate relatively few Japanese people exercise regularly (e.g. in 2016, < 10% of women and < 25% of men ages 20–39 exercised at least twice per week).^31^ Qualitative and quantitative theory-based research is needed to develop interventions to motivate Japanese people with SCI to exercise. Another barrier is that Japanese fitness centers tend to be less focused on improving clients’ strength and fitness and more oriented towards providing luxurious spaces for relaxation and entertainment, particularly for affluent older adults.^30^ It may be necessary to create new, dedicated exercise spaces for people with SCI.

In contrast to Japan, Korea’s rates of exercise participation in the general population are similar to rates reported in western countries.^32^ Interestingly, whereas approximately 50% of adults who live with a SCI in western countries do not participate in any leisure-time physical activity (LTPA; e.g.^33,34^), a small study (n=79) reported just 4% of Koreans with SCI did no LTPA whatsoever.^35^ Together, these data suggest exercise motivation may be less of a concern for Koreans with SCI. However, given the challenges faced by people with SCI to stay physically active long-term (e.g. secondary health complications, financial and transportation barriers),^36^ efforts will need to be directed toward supporting people in maintaining their exercise habits. Clearly, understanding country- and context-specific influences on exercise is an important next-step toward developing resources and interventions to support exercise among people living with SCI in Indonesia, Japan, Korea and Thailand.

When developing resources to support the implementation of the SEG-SCI,^1^ it will also be important to consider the preferences of people using the guidelines, such as their preferred types of exercise activities and the meanings people ascribe to exercise. Our review of the local SCI research showed that some of the activities used in SCI research interventions were unique to a particular country (e.g. a mat-based exercise program used in a Korean study; see Kwak *et al.* in Supplementary File 2). Also, many of the studies identified through the Indonesian-language literature search referred to exercise as *latihan*, which means “spiritual exercise”.^16^ In some cultures, the spiritual or psychological benefits of exercise may be considered just as valuable (if not more so) than the physical benefits.

### Project strengths and limitations

This project has provided evidence-based support for the use of the SEG-SCI^1^ in four Asian countries, as well as translations of the guidelines into four languages. Our collaborative, international processes for culturally validating and translating the SEG-SCI^1^ provides a model for those who want to adopt the guidelines in other regions.

Some limitations should be noted. First, our conclusions are limited by the research evidence available. As with the SCI exercise training literature in general,^6^ the reviewed studies included very few participants over the age of 65, relatively few women (some studies only included men), and focused predominately on people with chronic SCI. Therefore, our conclusions may not generalize to the entire SCI population. Likewise, because of the limited evidence base, we were not able to test all possible predictions associated with the SEG-SCI.^1^ For instance, we could not test the effects of a strength-training prescription that did not meet the SEG-SCI^1^ recommendation, on muscular strength. Second, validation (of models, measures, etc.) is an ongoing process that should involve multiple methods and approaches. It will be important for researchers and clinicians in these four countries to test the SEG-SCI^1^ and document their outcomes. Third, our project involved four countries who have a pre-existing partnership. While we consider it a strength of our project that we examined research from four Asian countries simultaneously, it might also be considered a weakness that other countries were not involved. Given limited resources, it was not possible to expand the scope of the project to include other countries.

## Conclusion

Based on the available local research evidence, we recommend that the Scientific Exercise Guidelines for Adults with Spinal Cord Injury^1^ be adopted for use in Indonesia, Japan, Korea and Thailand. The cultural validation and translation of the SEG-SCI^1^ for use in these countries is an important step towards establishing a consistent exercise prescription for SCI research around the world. The use of a consistent exercise prescription in SCI exercise training studies can advance knowledge regarding the dose of exercise needed to achieve specific health and fitness outcomes. The SEG-SCI^1^ can also be used in local clinical and community settings. However, evidence-based resources are needed to support implementation of the SEG-SCI^1^ in these contexts. Development of these resources will be aided by local research on barriers and facilitators to guideline implementation, and factors that influence exercise participation among people with SCI such as exercise preferences and the meanings ascribed to exercise.

## Supplementary Material

Supplemental Material
