# Supplementary material for: Cultural validation and language translation of the scientific SCI exercise guidelines for use in Indonesia, Japan, Korea, and Thailand
Source: J Spinal Cord Med. 2021 Jul 6;45(6):821–32. doi: 10.1080/10790268.2021.1945857 (PMC9661985; doi:10.1080/10790268.2021.1945857)
Supplement: Supplemental Material [file YSCM_A_1945857_SM8537.zip › Supplementary File 1 Databases and Search Strategies.pdf]

Supplementary File 1 Databases and Search Strategies

English

Databases:
MedLine (Ovid)
Embase
CINAHL
PsycInfo
SportDiscus

Filter 2016-2019

Keywords for SCI	brown-sequard syndrome<br>central cord syndrome<br>myelitis<br>myelopathy<br>paraplegia<br>spinal cord diseas*<br>spinal cord impair*<br>spinal cord injur*<br>spinal cord lesion<br>spinal cord transection<br>spinal impairm*<br>spinal injur*<br>spinal lesion<br>spinal transection<br>spine impairm*<br>spine injur*<br>spine lesion<br>spine transection<br>tetraplegia<br>exp Paraplegia/<br>Quadriplegia/<br>exp Spinal Cord Injuries/<br>Myelitis/<br>Myelitis, Transverse/<br>Spinal Cord Diseases/<br>Spinal Cord Compression/<br>exp Spinal Injuries/
Keywords for PA	aerobic capacity<br>Aerobic Exercise<br>endurance exercise<br>endurance training<br>exercise conditioning<br>exercise prescription<br>exercise therapy<br>exercise train*<br>exercise<br>motor activity<br>physical education<br>resistance training

	sport<br>strength training<br>exp Exercise Test/<br>Motor Activity/<br>exp Exercise/<br>exp Sports/<br>exp Muscle Strength/<br>exp Exercise Therapy/<br>exp Exercise Movement Techniques/<br>exp Physical Education and Training/<br>exp Physical Fitness/<br>exp Physical Endurance/

Indonesia

Databases:
Google Cendekia (scholar.google.co.id)
Garuda (garuda.ristekdikti.go.id)

Keywords for SCI	• Synonyms for SCI (e.g. **spinal cord injury**, SCI, spinal cord lesion, spine injury, quadriplegia, paraplegia)<br>• Common non-traumatic causes of SCI (myelitis, myelopathy, spinal cord disease)<br>• The SCI syndromes that American Spinal Injury Association (ASIA) recognizes (Brown-Sequard, cauda equine syndrome, central cord syndrome, anterior cord syndrome, conus medullaris syndrome).[1]
Keywords for PA	Exercise<br>**Latihan**<br>exercise conditioning<br>exercise prescription<br>exercise therapy<br>exercise training<br>motor activity<br>physical activity<br>fitness<br>physical fitness<br>physical education and training<br>physical education<br>sport<br>strength training<br>resistance training<br>aerobic capacity<br>aerobic exercise<br>endurance<br>endurance training<br>endurance exercise

Japan

Databases:
医中誌
CiNii

Keywords for SCI	Synonyms for SCI (脊髄損傷、頸髄損傷、腰髄損傷、胸髄損傷、仙髄損傷、頚椎損傷、胸椎損傷、腰椎損傷、脊椎損傷、四肢麻痺、対麻痺)<br>Common non-traumatic causes of SCI (脊髄炎、脊髄症、脊髄疾患)<br>The SCI syndromes that American Spinal Injury Association (ASIA) recognizes (ブラウン-セカード、馬尾症候群、中枢症候群、前索症候群、延髄症候群).[1]
Keywords for PA	運動<br>訓練<br>運動調節<br>運動処方<br>運動療法<br>運動トレーニング<br>運動活動<br>身体活動<br>フィットネス<br>基礎体力<br>体力<br>体育とトレーニング<br>体育<br>スポーツ<br>筋力トレーニング<br>ウエイトトレーニング<br>抵抗訓練<br>有酸素能力<br>エアロビック<br>有酸素運動<br>耐久性<br>持久力トレーニング<br>持久力運動

Korea

Databases:
RISS: Research Information Service System
KISS: Korean studies Information Service System
DBpia: DataBase Periodical Information Academic

Keywords for SCI	• Synonyms for SCI (척수장애인, 척수손상, 경수손상, 사지마비, 하지마비)<br>• Common non-traumatic causes of SCI (척수염, 척수병증, 척수질환)<br>• The SCI syndromes that American Spinal Injury Association (ASIA) recognizes (브라운시쿼드, 마미 증후군, 마미 신경총 증후군, 중심성 척수 증후군, 척수 증후군, 척수 원추 증후군, 척수 원뿔 증후군, Brown-Sequard*) / **16 Keywords**
Keywords for PA	운동치료<br>운동중재<br>운동처치<br>운동능력<br>신체활동<br>운동교육<br>스포츠<br>트레이닝<br>근력운동<br>근력훈련<br>저항운동<br>지구력 운동<br>지구력 훈련<br>유산소 운동<br>유산소성 능력<br>유산소성 파워<br>심폐능력<br>활동수준**/ 18 Keywords**

Brown-Sequard*: English searching

Thailand

Databases:
Thai-Journal Citation Index(TCI)
Thai Journal of Nursing Council
Journal of the Medical Association of Thailand
ASEAN Journal of Rehabilitation Medicine (Journal of Thai Rehabilitation Medicine
Thai Journal of Physical Therapy
PubMed 2016-2019

Keywords for SCI*	• Synonyms for SCI (e.g. spinal cord injury, SCI, spinal cord lesion, spine injury, quadriplegia, paraplegia)<br>• Common non-traumatic causes of SCI (myelitis, myelopathy, spinal cord disease)<br>• The SCI syndromes that American Spinal Injury Association (ASIA) recognizes (Brown-Sequard, cauda equine syndrome, central cord syndrome, anterior cord syndrome, conus medullaris syndrome).[1]
Keywords for PA**	exercise<br>exercise conditioning<br>exercise prescription<br>exercise therapy<br>exercise training<br>motor activity<br>physical activity<br>fitness<br>physical fitness<br>physical education and training<br>physical education<br>sport<br>strength training<br>resistance training<br>aerobic capacity<br>aerobic exercise<br>endurance<br>endurance training<br>endurance exercise
