# Supplementary material for: Cultural validation and language translation of the scientific SCI exercise guidelines for use in Indonesia, Japan, Korea, and Thailand
Source: J Spinal Cord Med. 2021 Jul 6;45(6):821–32. doi: 10.1080/10790268.2021.1945857 (PMC9661985; doi:10.1080/10790268.2021.1945857)
Supplement: Supplemental Material [file YSCM_A_1945857_SM8537.zip › Supplementary File 2R Studies included in the review .docx]

**Supplementary File 2.** Data extracted^†^ from relevant studies conducted in Indonesia, Japan, Korea and Thailand (1980-2019).

| **Study**  **Country and Publication Language**  **Design**  **Quality score**  **Level** | **Acute or Chronic SCI** | **N (M/F)** | **Participants** | **Intervention and Control** | **Relative intensity** | **Duration**  **(min)** | **Frequency (times /week)** | **Intervention period (weeks)** | **Cardio-respiratory fitness** | **Power output** | **Muscle strength** | **Body com-position** | **Cardiovascular risk factors** | **Bone health** | **Adverse events (AE) related to intervention^1^** |
| --- | --- | --- | --- | --- | --- | --- | --- | --- | --- | --- | --- | --- | --- | --- | --- |
|  |  |  |  |  |  |  |  |  |  |  |  |  |  |  |  |
| **Fukuoka et al. 2006**  **Japan, English**  Pre-post  D&B=15  *Level 4* | Chronic | 8 (7/1) | Age: 47±8 y  TSI: NR  Lesion**:** AIS B; T7-L1 | Wheelchair exercise | 50% HRR | 30 min | 3 | 8.6 | Improv.  VO2peak arm crank: ↑*  VO2 kinetics:↑* | No Improv. |  |  |  |  | NR |
| **Higuchi et al.**  **2009**  **Japan, Japanese**  Pre-post  D&B=16  *Level 4* | NR | 20 (16/4) | Age: Intervention (n=6) 51.7±10.9 y  Lesion**:** C5-L1  C1: Sportif (n=8) 43.0±9.3 y  Lesion**:** C6-T12  C2: Non-exercise(n=6)  58.3±13.0 y  Lesion**:** C6-C8 | One of wheelchair exercise, walking exercise, passive bicycle exercise | 50%  VO_2max_ | 30min | >1 | 12 |  |  |  | Improv.  body weight: ↔  VF(Visceral fat): ↔  Waist circumference: ↓* | Improv.  TC: ↔  TG : ↔  HDL-C: ↔  LDL-C: ↔  Atherosclerosis Index: ↓*  Blood glucose level: ↔  insulin: ↔  A1C: ↓*  Leptin: ↓*  Adiponectin: ↔ |  | NR |
| **Horiuchi et al. 2017**  **Japan, English**  Pre-post  D&B=14  *Level* ***4*** | Chronic | 9  (9/0) | Age: 38±10 (26-55) y  TSI: 16±7 (7-28) y  Lesion: AIS A-B; T8-L1 | Arm-cranking exercise | Progressive over weeks 50-70% HR reserve | 60 min (2 bouts of 30 min with 10-min rest) | 4 | 10 | Improv.  VO2peak arm crank: ↑*  Peak V_E_ ↑*  Peak HR ↔ |  | No Improv.  Handgrip strength: ↔ | Improv.  Body Mass: ↓*  Waist circumference: ↓* | Improv.  SBP: ↓*  DBP: ↔  Blood glucose: ↔  Hemoglobin A1c: ↔  Total cholesterol: ↔  TG: ↓*  HDL-C: ↔  LDL-C: ↔  PAI-1: ↓*  Fibrinogen: ↔ |  | NR |
| **Jung et al. 2014**  **Korea, English**  Pre-post  D&B=21  Level 3 | Acute | 20 (12/8) | Age: 42±11 and 51±9 for subgroups  TSI: 38±18 and 38±15 weeks  Lesion**:** AIS B-D; C8-L5 | E1: Aquatic including sitting, standing and walking  E2: As E1, but overground | NR | 60 min | 3 | 8 | Improv. (E1 and E2)  FER  E1:↑*  E2:↑*  FVC  E1:↑*  E2: ↔  FEV1  E1:↑*  E2: ↔  FEV1 to FVC ratio  E1:↑*  E2: ↔ |  |  |  |  |  | NR |
| **Kim et al. 2014**  **Korea, English**  Pre-post  D&B=15  *Level 4* | Chronic | 12 (10/2) | Age: 36±12 (16-45) y  TSI: 11±6 (5-24) y  Lesion**:** AIS A-C; C6-L1 | Hybrid FES rowing with interval protocol | 5-min sets with 30-s rest intervals while reaching at least 70% HRpeak | 32.5 min (excl. rest intervals, warm-up and cool-down) | 5 | 6 | No Improv.  VO2peak arm crank: ↔ |  | Improv. shoulder strength: ↑*  elbow strength: ↑* | Improv.  Lean mass: ↑*  Body fat %: ↓*  BMI: ↔  Waist circumference: ↔ |  |  | NR |
| **Kim et al. 2015**  **Korea, English**  RCT  PEDro=5  *Level 2* | Chronic | 15  (9/6) | Age: 33±6 (22-46) y  TSI: 7±4 (2-16) y  Lesion**:** AIS A-B; C5-T11 | E: Stationary hand cycling using wheelchair with add-on arm crank and game-like software  C: No exercise intervention | Progressive over weeks: 70 to 80% HRpeak or 5 to 7 on CR10 scale | 36 min (12 sets of 3-min exercise interspersed with 1-min rest) total of 60 min incl. warm-up and cool-down | 3 | 6 | Improv.  VO2peak arm crank: ↑*# |  | Improv.  Elbow flexion: ↑*#  Elbow extension: ↑*#  Shoulder abduction; ↑*#  Shoulder adduction: ↑*#  Shoulder flexion: ↑*#  Shoulder extension: ↑*# | Improv.  Lean mass: ↔  Body fat %: ↔  BMI: ↓*#  Waist circumference: ↓*# | Improv.  HOMA-IR: ↓*#  Fasting insulin: ↓*#  HDL: ↑*  LDL: ↔  TC: ↔  Triglycerides: ↔  Glucose: ↔ |  | NR |
| **Kim et al. 2019**  **Korea, English**  RCT  PEDro=6  *Level 1* | Chronic | 19  (12/7) | Age: 36.8±6.9  (23-53) y  TSI: 9.23  (2-27) y  Lesion: AIS A,B,C; C4-L1 | E: Resistance, circuit, and arm ergometry or hand-cycling  C: Standard Care | Progressive over weeks 65-85% max HR or Borg 4 to 8 on 10-point scale | 60 min (25min warm-up [5min joint exercises, 15min arm ergometer, 5min stretching], resistance training [1-3 sets, 10-20 reps], circuit training [1-2 sets], aerobic training [10-20 min] | 3 | 6 | Improv.  VO2peak: ↑* NS compared to control group |  | Improv.  Elbow flexion: ↑*#  Elbow extension: ↑*#  Shoulder abduction; ↑*#  Shoulder adduction: ↑*#  Shoulder flexion: ↑*#  Shoulder extension: ↑*# | Improv.  Waist circumference: ↓#  BMI: ↓*  Body fat%: ↓*  Lean Mass: ↔ | Improv.  HOMA-IR: ↓*#  Fasting insulin: ↓*#  HDL-C: ↑*#  LDL-C: ↔  TC: ↔  Glucose: ↔ |  | NR |
| **Kondo et al.**  **1995**  **Japan, Japanese**  Pre-post  D&B=14  *Level 4* | Chronic | 5  (5/0) | Age: 22.8±4.2 y  Lesion: C6 | Swimming training | 60-90%HRR | 40-60min | 3 | 12 | Improv.  VC: ↑*  FEV1: ↑*  expiratory reserve volume: ↑*  inspiratory reserve volume: ↔  FVC: ↑*  expiratory flow rate: ↑*  V25%/Height: ↑*  Maximal ventilation volume: ↑*  Respiratory Rate: ↓*  Tidal Volume: ↑* |  |  |  |  |  | NR |
| **Kubota 2019**  **Japan, English**  Pre-post  D&B=17  Level 4 | Chronic | 8  (4/4) | Age: 60.9 ± 10.2 (43-77) y  Lesion: Cervical and thoracic OPLL; AIS D; Stenosis from T2/3-T11/12 | Hybrid Assisted Limb (HAL) walking using All-In-One Walking Trainer | NR | 15-20 min | 2-3 | 4-5 |  | Improv. WISCI-II: ↑  10MWT: Gait speed: ↑*  Step length: ↑*  Cadence: ↑* | Improv.  AIS motor score (lower extremities): ↑*  FIM motor: ↑* |  |  |  | No serious or other AE |
| **Kulsomboon et al**  **1993**  **Thailand, Thai**  Pre-post  D&B=15  *Level 4* | NR | 10  (6/4) | Age: NR  TSI: NR  Lesion**:** AIS A; T8-T12 | Arm cranking | 70-80% peak heart rate | 20-30 min | 3-5 times per week | 3 weeks | Improv.  Peak SBP ↓* Resting DBP ↑*  peak DBP↑*  Resting HR↔  Peak RPP ↔ Peak RPE ↔ | No improv. |  |  |  |  | No serious AE |
| **Kwak et al.**  **2014**  **Korea, Korean**  Pre-post  D&B=18  Level 4 | Acute | 14  (14/0) | Age: 31-40, n=6; 41-50, n=3; 51-60, n=5  Lesion: T1-T6, n=5;  T7-T12, n=9 | E1: n=7 10-15 reps of 3 sets of  mat exercise program  (Dips on push-up bar, Pull-up, Push-ups on marjaryasana position)  E2: n=7 Sling exercise Program (Supine Pull-ups, Dips, Push-ups) | 5-min preparing  10 sec rest between exercise  RPE 3-4 | 30-40 min | 3 | 8 |  |  | Improv.(E1 and E2)  MMT strength  E1. ↑  (Shoulder-flexion / Elbow- extension / Wrist-extension),  E2. ↑  (Shoulder-flexion, extension, abduction, aduction/ Elbow-flexion, extension, supination, pronation/ Wrist-flexion, extension),  CUE  E1. ↑  E2. ↑  SCIMⅢ  E1. ↑  E2. ↑ |  |  |  | No AE |
| **Okawara et al. 2020**  **Japan, English**  Pre-post  D&B=17  *Level* ***4*** | Chronic | 20  (15/5) | Age: 43±17  TSI: 80±129 months  Lesion: AIS A,B,C,D; C-L | Hybrid Assisted Limb (HAL) with Body weight supported treadmill training (BWSTT) | Between 0.5 and 2.5km/h; No incline. | 60 min, with up to 20 min rest time | 2-5 | 20 sessions |  | Improv.  WISCI-II: ↔  10MWT: ↓*  Gait speed: ↑*  #of Steps: ↓*  2MWT:  Distance: ↑*  TUG: ↓* | Improv.  Berg Balance Scale (BBS): ↑*  ADLs: Barthel Index: ↔  FIM Functional Independence Measure: ↔ |  |  |  | No AE |
| **Panthong et al.**  **2011**  **Thailand, Thai**  Pre-post  D&B=16  *Level 4* | NR | 15  (12/3) | Age: 34.5±12.0 y  TSI: NR  Lesion**:** Lumbar level and below; incomplete and complete SCI (specific lesions + AIS NR) | core stabilization exercise program | NA | at least 20- 40 minutes | 5 | 4 |  |  | Improv.  abdominal and back muscles strength↑* |  |  |  | NR |
| **Rachmawati et al.**  **2004**  **Indonesia, Indonesian**  Pre-post  D&B=12  *Level 4* | Chronic | 23 (19/4) | Age: 34.74 ± 7.63 y  TSI: 12.22 ± 7.32 y  Lesion: below T6 (AIS A/B/C T7-L3) | Arm ergometer | 70-85% HRmax | 25 min | 3 | 6 | Improv.  VO2max ↑*  V_E_ ↑* |  |  |  |  |  | NR |
| **Yim 1993**  **Korea, English**  Pre-post  D&B=18  *Level 4* | Chronic | 11 (11/0) | Age: 31±8 y  TSI: 2±1 y  Lesion**:** AIS A; T8-T12 | Wheelchair ergometry | Up to 80% age-predicted HR | 30 min (3 bouts of 10 min with 5-min rest) | 2 | 5 | No Improv.  Rest HR: ↔  FVC: ↔  FEV: ↔  PEF: ↔ |  | Improv.  Isokinetic peak torque  Shoulder flexion:↑*  Shoulder extension: ↔  Elbow flexion: ↔  Elbow extension: ↔ |  |  |  | NR |

If not indicated otherwise, interventions were in supervised rehabilitation settings or did not specify the setting.
^†^Included data, as shown in the table, on:

- study design;
- participant characteristics, i.e. sample size, demographics, lesion characteristics;
- PA intervention conditions, i.e. PA type, frequency, intensity, duration, progression, and intervention length;
- control conditions, i.e. if and what intervention was provided to the control group;
- outcomes, i.e. outcome category, outcome measures, significant improvement or not in each outcome measure within or between groups;
  - “Improv” = one or more of the outcome measures within a column showed a significant improvement;
  - “No Improv” = none of the outcome measures within a column showed a significant improvement;
  - * indicates a significant pre-post improvement in the outcome (for studies with a control group, no difference compared to the control group)
  - # indicates a significant improvement compared to the control group
  - *# indicates a significant pre-post improvement *and* a significant improvement compared to the control group

Abbreviations: ADL = Activities of daily living; AE = adverse event; AIS = American Spinal Injury Impairment Scale; BWSTT = bodyweight-supported treadmill training; C = control group; CR10 = Borg’s 10-point category-ratio scale; CUE=Capabilities of Upper Extremities Instrument; DBP = diastolic blood pressure; D&B = Downs and Black score; E = exercise group; FER = forced expiratory ratio; FES = functional electrical stimulation; FEV1 = forced expiratory volume in one second; FVC = forced vital capacity; HDL = high-density cholesterol; HR = heart rate; HRpeak = peak heart rate; HRR = heart rate reserve; Improv. = improvement; LDL = low density cholesterol;

MMT = manual muscle test score; NR = not reported; NS = not significant; PAI-1=Plasminogen Activator Inhibitor 1; PEDro = Physiotherapy Evidence Database tool; PEF = peak expiratory flow; RPE = ratings of perceived exertion; RPP = rate pressure product; SBP = systolic blood pressure; SCI = spinal cord injury; TC = triglycerides; TSI = time since injury; TUG: Timed Up and Go; VC = vital capacity; VO2 = oxygen uptake; VO2peak = peak oxygen uptake on maximal graded exercise test (or similar); V_E_ = pulmonary ventilation; WISCI = Walking Index for Spinal Cord Injury; 10MWT = 10-m walk test

**References**

1. Fukuoka Y, Nakanishi R, Ueoka H, Kitano A, Takeshita K, Itoh M. Effects of wheelchair training on VO2 kinetics in the participants with spinal-cord injury. Disability and rehabilitation. Assistive technology 2006; 1(3): 167-74.
2. Higuchi Y, Sakuma H, Nakazawa K, Ishii N, Koinuma T, Iwaya T. The influence of exercise intervention for spinal-cord-injury persons with metabolic syndrome. J. Physical Medicine 2009; 20(3): 269-274.
3. Horiuchi M, Okita K. Arm-Cranking Exercise Training Reduces Plasminogen Activator Inhibitor 1 in People With Spinal Cord Injury. Arch Phys Med Rehabil 2017; 98(11): 2174-2180.
4. Jung J, Chung E, Kim K, Lee B-H, Lee J. The effects of aquatic exercise on pulmonary function in patients with spinal cord injury. J Phys Ther Sci 2014; 26(5): 707-709.
5. Kim DI, Park DS, Lee BS, Jeon JY. A six-week motor-driven functional electronic stimulation rowing program improves muscle strength and body composition in people with spinal cord injury: a pilot study. Spinal Cord 2014; 52(8): 621-4.
6. Kim DI, Lee H, Lee BS, Kim J, Jeon JY. Effects of a 6-Week indoor hand-bike exercise program on health and fitness levels in people with spinal cord injury: a randomized controlled trial study. Archives of Physical Medicine and Rehabilitation 2015; 96(11): 2033-2040.
7. Kim DI, Taylor JA, Tan CO, Park H, Kim JY, Park SY et al. A pilot randomized controlled trial of 6-week combined exercise program on fasting insulin and fitness levels in individuals with spinal cord injury. European spine journal : official publication of the European Spine Society, the European Spinal Deformity Society, and the European Section of the Cervical Spine Research Society 2019; 28(5): 1082-1091.
8. Kondo T, Kobayashi I. Effects of swimming training on respiratory function in patients with quadriplegia due to cervical spinal cord injury. The Journal of Rehabilitation Sports 1995; 14(1): 44-47.
9. Kubota S, Abe T, Kadone H, Shimizu Y, Funayama T, Watanabe H et al. Hybrid assistive limb (HAL) treatment for patients with severe thoracic myelopathy due to ossification of the posterior longitudinal ligament (OPLL) in the postoperative acute/subacute phase: A clinical trial. J Spinal Cord Med 2019; 42(4): 517-525.
10. Kulsomboon W, Khunphasee A, Teeranet K, Ing-Aram R, Khunadorn F. Cardiovascular Response of Paraplegics on Arm Cranking Exercise. J Thai Rehabil 1993; 3(2): 12-17.
11. Kwak SK, Song YE, Kim BW, Kim CH, Kang EB. The effect of 8 weeks sling exercise program on upper limb muscle strength CUE and SCIM Ⅲ in spinal cord injury patients. The Korea Journal of Sport 2014; 12: 415-426.
12. Okawara H, Sawada T, Matsubayashi K, Sugai K, Tsuji O, Nagoshi N et al. Gait ability required to achieve therapeutic effect in gait and balance function with the voluntary driven exoskeleton in patients with chronic spinal cord injury: a clinical study. Spinal Cord 2020; 58(5): 520-527.
13. Panthong P, Thongauam S. The Outcomes of Abdominal and Back Muscle Exercise in Spinal Cord Injury Patients at Maharat Nakhon Ratchasima Hospital. Nakhon Ratch Med Bull 2011; 35: 157-162.
14. Rachmawati MR. Effect of aerobic exercise on cardiorespiratory capacity in spinal cord injured patients. J Kedokter Trisakti 2004; 23(1): 15-20.
15. Yim SY, Cho KJ, Park CI, Yoon TS, Han DY, Kim SK et al. Effect of wheelchair ergometer training on spinal cord-injured paraplegics. Yonsei medical journal 1993; 34(3): 278-86.
