# Supplementary material for: Cultural validation and language translation of the scientific SCI exercise guidelines for use in Indonesia, Japan, Korea, and Thailand
Source: J Spinal Cord Med. 2021 Jul 6;45(6):821–32. doi: 10.1080/10790268.2021.1945857 (PMC9661985; doi:10.1080/10790268.2021.1945857)
Supplement: Supplemental Material [file YSCM_A_1945857_SM8537.zip › Supplementary File 4 Comments on Back Translations.pdf]

Supplementary File 4. Comments on the backward translations of the Scientific SCI Exercise Guidelines.

Indonesia

	Original Text	Back Translation	Comments
Title	Scientific exercise guidelines for adults with spinal cord injury	Scientific guideline for adult spinal cord injury patient exercise	• the exercises are for the SCI patient (Scientific guideline for adults spinal cord injury **patient's exercise**).
Paragraph 1	**These** exercise guidelines **provide** minimum thresholds for achieving the following benefits:<br>**improved cardiorespiratory** fitness and muscle strength<br>improved **cardiometabolic health**	**This** exercise guideline **give** the minimum threshold to achieve benefits of:<br>**restoring cardio-pulmonary** fitness and muscle strength<br>restoring **the health of cardiac metabolism**	• The original version uses plural form, in back translate use single form. In bahasa Indonesia there were no single or plural form for showing pronoun, so it could make a difference in back translation but it still have the same meaning.<br>• Provide and give have the same meaning in bahasa Indonesia.<br>• Improved in bahasa Indonesia can be translated become "meningkatkan" (=increase, improve) or "memperbaiki" (= restoring, improve). The translator use word "memperbaiki" in bahasa Indonesia, so the back translation become restoring.<br>• The word respiratory, if translate to bahasa Indonesia, has a close meaning to breathing system. In term of cardiorespiratory fitness, the translation into bahasa Indonesia want to show the fitness of cardiac and lungs system. So when it back translate to English, become Cardiopulmonary.<br>• The word metabolic when translated into bahasa Indonesia became "metabolism", which is the same as metabolism when it transalate back into English, so the back translation become: the health of cardiac metabolism.
Paragraph 2	The guidelines should be achieved above and beyond the incidental physical activity one might accumulate in the course of daily living.	This guideline is expected to exceed routine physical activity which varied and accumulated throughout the day.<br>Adults are suggested to participate routinely in a	• The difference meaning of the sentences especially in the last word. Words "Reasonably achievable" were translated into "terjangkau" which has meaning can be reach, also use for affordable. The translator used the

	Adults are encouraged to participate routinely in exercise modalities and contexts that are sustainable, enjoyable, safe, and **reasonably achievable**.	regular, fun, safe, and **affordable exercise**.	word affordable for the back translation.
Paragraph 3	These guidelines are **appropriate** for adults (aged 18-64) with chronic spinal cord injury (**at least** one year **post onset**), **neurological level of injury C3** and below), from traumatic or non-traumatic causes, including **tetraplegia and paraplegia**, **irrespective** of sex, race, ethnicity or socio-economy status.	This guideline is **suitable** for chronic spinal cord injury patients (one year **after onset** in **minimum**, with **a level of injury on cervical 3** or below), aged between 18 to 64 years old, which can be caused by traumatic or non-traumatic event, including **weakness of four limbs and weakness of both lower limbs,** with **no regard to** sex, race, ethnicity, nor socio-economy status.	• For the first and second phrases, overall have the same meaning but with different forms of sentence.<br>• Words tetraplegia and paraplegia were translated into Bahasa Indonesian with the explanation what tetraplegia and paraplegia are. Because for non-medical population, almost do not know the meaning of tetraplegia or paraplegia. So the back translation become weakness of limbs.<br>• Neurological level of injury C3 were translated into level of injury on cervical 3. We think *level of injury of cervical 3* have an ambigous meaning, it can be 3rd cervical spine or 3rd cervical nerve.<br>• For this back translation it should be "**level of injury on 3rd cervical nerve**".
Paragraph 4	Before starting an exercise programme, adults with SCI should consult with a health professional who is knowledgeable in the types and amounts of exercise appropriate for people with SCI.<br>Individuals with a cervical or **high thoracic injury** should be aware of the signs and symptoms of autonomic dysreflexia during exercise.	Before starting this exercise program, spinal cord injury patients must consult to professional health care provider with adequate knowledge about the type and amount of exercise need to be done according to the patient's condition.<br>Someone with cervical and **upper back injury**, must be aware about the signs and symptoms of autonomic dysreflexia during exercise.	• In this paragraph, the major different was the word "high thoracic injury" became "upperback trauma" in the back translation.<br>• The meaning of thoracic in Bahasa Indonesia associated with chest cavity or back part/bones of the body at the upper region.<br>• The first translation from original version into bahasa Indonesia, the word high thoracic injury was translated into "trauma tulang punggung atas" (= trauma at upper back/bone parts). So when it translated back into english become "upperback taruma / upperback injury".
Paragraph 5	For adults who are not already exercising, it is appropriate to start with smaller amounts of exercise and gradually increase duration, frequency, and intensity, as a progression	If the patient has never exercise before, it is suggested to start the exercise in a gradual manner. Start with a light portion and gradulally increase in the duration, frequency and intensity of the exrcise until reaching	• Generally yhe sentences have the same meaning with different forms of sentences. This might be caused by the diffrence of tenses between english and bahasa Indonesia.

	toward meeting the guidelines.<br>Doing exercise below the recommended level may or may not bring small changes in fitness or **cardiometabolic health**.	to what the guideline suggest.<br>Exercise done below the level recommended may or may not give small changes in fitnes or health of **cardiac metabolism**.	• For word "cardiacmetabolic health" become "cardiac metabolism" the explanation same as before in first paragraph: the word metabolic when translated into bahasa Indonesia became "metabolisme" which is same as metabolism when it transalate back into English, so the back translation become: the health of cardiac metabolism.
Paragraph 6	The risks associated with these guidelines are minimal when managed in consultation with health care professional who is knowledgeable in spinal cord injury.	This guideline has minimal risk when it is done according to the direction of health professional with adequate knowledge on spinal cord injury	• Overall, the meaning of the back translation almost the same as the original version.
Paragraph 7	The guidelines may be appropriate for individuals with a SCI less than 12 months post-onset, aged 65 years or older, or living with comorbid conditions.<br>There is currently insufficient scientific evidence to draw firm conclusions about the risks and benefits of the guidelines for these individuals.<br>These individuals should consult a **health care provider** prior to beginning an exercise programme.	These guidelines may be appropriate for individuals with less than 12 months spinal cord injury onset, aged 65 years old above, or individuals with other comorbidities.<br>But there are not enough scientific evidence to support the conclusion about the risk and benefit for the individual groups mentioned above.<br>They are suggested to consult with the **guideline provider** before starting this exercise program.	• Generally the meaning of overall sentences in this part have the same meaning, with different structure of sentences.<br>• Health care provider in original version were translated into bahasa Indonesia specifically as provider who make this guideline. So it became guideline provider when translated back into English version.
Paragraph 8	Exceeding these exercise guidelines would be expected to yield additional cardiorespiratory fitness and muscle strength and cardiometabolic health benefits.<br>However, there are insufficient data to comment on the risks associated with a person with SCI **exceeding these guidelines.**	The exercise exceeding these exercise guidelines, is hoped can give additional benefit in cardio-pulmonary fitness and muscle strength and the health of cardiac metabolism.<br>But there is not enough data found reviewing about the risk of spinal cord injury patients who practice **exceeds these guidelines.**	• In the first sentence of this paragraph, generally has the same meaning.<br>• The second sentence, the "back translate 1" have different at the last words. However after back translation into bahasa Indonesia and translated back again into english (back translate 2), the meaning are same as original version.
Subtitle Fitness	For **cardiorespiratory** fitness and muscle strength benefits, adults with SCI should engage in at least:	To get the benefit on **cardio-pulmonary** fitness and muscle strength, adults with spinal cord trauma have to at least do:	• The main differences in this part are the translation of "each major functioning muscle group" become "shoulder and hips muscles".<br>• General population in Indonesia do not know the

	20 minutes of moderate to vigorous intensity aerobic exercise, 2 times a week<br>3 sets of strength- training exercises **for each major functioning muscle group**, at moderate to vigorous intensity, 2 times a week	20 minutes of fitness exercise starting with moderate intensity to high intensity, 2 times a week<br>3 sets of muscle strengthening **for shoulder and hip muscles** starting with moderate intensity to high intensity, 2 times a week.	meaning of major functioning muscle group, so the translator specifically gives examples of the specific examples of major functioning muscle group (which are shoulder and hip muscles). So when it translated back into English version, the words **major functioning muscle group** become **shoulder and hip muscles.**
Subtitle Cardiometabolic health	For cardiometabolic health benefit, adults with SCI are suggested to engage in at least:<br>30 minutes of moderate to vigorous intensity aerobic exercise, 3 times a week	To get the benefit for cardiac metabolism health, adult spinal trauma patients are suggested to at least do:<br>30 minutes of fitness exercise starting with moderate intensity to high intensity,<br>3 times a week.	• Overall the back translations have the same meaning as original version.<br>• For word "cardiacmetabolic health" become "cardiac metabolism" the explanation same as before in first paragraph: the word metabolic when translated into bahasa Indonesia became "metabolisme" which is same as metabolism when it transalate back into English, so the back translation become: the health of cardiac metabolism.

Japan

	Original Text	Back Translation	Comments
Title	Scientific exercise guidelines **for adults** with spinal cord injury	Scientific exercise **for those** with a spinal cord injury	• **Since most people with SCI are adults in Japan, it is not necessary to specify 'for adults'**
Paragraph 1	These exercise guidelines provide minimum thresholds for achieving the following benefits:<br>• improved cardiorespiratory fitness and muscle strength<br>• improved cardiometabolic health	These **guidelines show the minimum level of exercise needed to gain the benefits stated below.**<br>**Improvement** of cardiopulmonary function and muscle strength<br>**Amelioration** of cardiovascular and metabolism	• As the title has already showed 'exercise guideline', the word 'exercise' has been removed in this phrase. Also, 'guidelines' is widely used in a Japanese context,<br>• The word 'thresholds' was too academic to use in this context and it was replaced with a easier word that is the minimum level of exercise.<br>• Either improve or increase can be used in a Japanese context.<br>• There is no equivalent expression of cardiometabolic health.
Paragraph 2	The guidelines should be achieved above and beyond the incidental physical activity	In these guidelines, people are recommended to do exercise which exceeds the amount of daily physical	• It was hard to translate the expression '**achieved above and beyond the incidental physical activity**

	one might accumulate in the course of daily living. Adults are encouraged to participate routinely in exercise modalities and contexts that are sustainable, enjoyable, safe and reasonably achievable.	activities. Adults are recommended to regularly participate in exercise in a sustainable, safe, enjoyable and reasonably achievable method.	・”encouraged”is much more likely to say ‘recommend’<br>• Exercise modalities can be simply translated into ‘exercise’
Paragraph 3	These guidelines are appropriate for adults (aged 18-64) with chronic spinal cord injury (at least one year post-onset, neurological level of injury C3 and below), from traumatic or non-traumatic causes, including tetraplegia and paraplegia, irrespective of sex, race, ethnicity or socio-economic status.	These guidelines are suitable for those adults (18-64 years old) with a chronic spinal cord injury (at least 1 year since the origin and development of the injury and under c3 in neurological injury level) that includes acroparalysis and paraplegia caused traumatically or atraumatically regardless of sex, race, ethnicity, social and economical status.	
Paragraph 4	Before starting an exercise programme, adults with SCI should consult with a health professional who is knowledgeable in the types and amounts of exercise appropriate for people with SCI. Individuals with a cervical or high thoracic injury should be aware of the signs and symptoms of autonomic dysreflexia during exercise.	Before a person with a spinal cord injury begins any exercise program it is necessary for her/him to consult with professional health providers who specialize in the method and amount of exercise that is suitable for people with spinal cord injuries. Those with severe injuries in their neck or chest need to pay attention to the signs and symptoms of autonomic dysreflexia while exercising.	• As mentioned above, most people with SCI are adults, so I it was not necessary to specify that the guidelines is for adults.
Paragraph 5	For adults who are not already exercising, it is appropriate to start with smaller amounts of exercise and gradually increase duration, frequency, and intensity, as a progression toward meeting the guidelines. Doing exercise below the recommended levels may or may not bring small changes in fitness or cardiometabolic health.	For those adults who haven’t begun exercising yet, it is suitable for them to start with light exercise and gradually increase the duration, frequency and intensity to meet the guidelines. If less than the recommended level of exercise is done, there is a chance that only small changes are made in the improvement of cardiopulmonary function and muscle strength or amelioration of cardiovascular and metabolism or there is a chance that no changes are made.	• In this context, the adults are subject and for the flow of information, adults were added intentionally.
Paragraph 6	The risks associated with these guidelines are minimal when managed in consultation with a health care professional who is	One can keep these risks related to the guidelines minimum if one manages it by consulting professional health providers who specialize in spinal cord injuries.	• ‘When’ phrase can be better to be translated into ‘if ‘phrase in Japanese.

	knowledgeable in spinal cord injury.		
Paragraph 7	The guidelines may be appropriate for individuals with a SCI less than 12 months post-onset, aged 65 years or older, or living with comorbid conditions. There is currently insufficient scientific evidence to draw firm conclusions about the risks and benefits of the guidelines for these individuals. These individuals should consult a health care provider prior to beginning an exercise programme.	There is a chance that the guidelines are suitable for those with a spinal cord injury who have had it less than 12 months, who are over the age of 65 or those who have comorbidity. Currently there is not enough scientific evidence to draw an incontrovertible conclusion about risks and benefits of the guidelines for those people. Those people need to consult with professional health providers before they start exercise programs.	
Paragraph 8	Exceeding these exercise guidelines would be expected to yield additional cardiorespiratory fitness and muscle strength and cardiometabolic health benefits. However, there are insufficient data to comment on the risks associated with a person with SCI exceeding these guidelines.	If one exercises beyond the exercise guidelines it is expected that one can gain further ameriolation in his/her cardiopulmonary function, muscle strength, cardiovascular and metabolism. However there is not enough data of the risks of exercise beyond the guidelines for people with spinal cord injury.	
Subtitle Fitness	Fitness<br>For cardiorespiratory fitness and muscle strength benefits, adults with SCI should engage in at least:<br>20 minutes of moderate to vigorous intensity aerobic exercise, 2 times a week<br><br>3 sets of strength-training exercises for each major functioning muscle group, at a moderate to vigorous intensity, 2 times a week	Amelioration of cardiopulmonary function and muscle strength<br><br>To improve cardiopulmonary function and muscle strength a person with a spinal cord injury needs the following exercises at minimum.<br><br>20 minutes moderate to intense cardio exercise, 2 times a week<br><br>3 sets of moderate to intense strength training of muscle groups responsible for major exercise	• The term ‘fitness’ represents the image of Gym (the place) in Japan and thus, ‘fitness’ was replaced with ‘cardiorespiratory fitness and muscle strength benefits.<br>・’should engage’ gives an impression of very strong instruction but the phrase that ‘it is necessary to’ become soft and polite<br>・It was hard to translate ‘for each major functioning muscle group’
Subtitle Cardiometabolic health	Cardiometabolic health<br>For cardiometabolic health benefits, adults with SCI are suggested to engage in at least:	Amelioration of cardiovascular and metabolism.<br><br>To improve cardiovascular and metabolism the following exercise is recommended at minimum for a	・Cardiometabolic health is translated into cardiovascular and metabolism<br>・the term ‘Vigorous’ is equivalent to the word ‘intense’ in Japanese

	30 minutes of moderate to vigorous intensity aerobic exercise, 3 times a week	person with a spinal cord injury.<br><br>30 minutes moderate to intense cardio exercise, 3 times a week	

Korea

	Original Text	Back Translation	Comments
Title	Scientific exercise guidelines for adults with spinal cord injury	Scientific Exercise Guideline for Adults with Spinal Cord Injury	• In case of the word 'guidelines' it can be singular or plural as there is no difference in Korean.
Paragraph 1	These exercise guidelines provide minimum thresholds for achieving the following benefits:<br>• improved cardiorespiratory fitness and muscle strength<br>• improved cardiometabolic health	This exercise guideline proposes a minimum baseline to achieve two following purposes.<br>• improve cardiopulmonary fitness and muscular strength<br>• improve metabolic fitness of cardiovascular system	• As 'cardiometabolic' has no equivalent term, 'improved cardiometabolic health' in the original text became 'metabolic fitness of cardiovascular system' in the back-translation.
Paragraph 2	The guidelines should be achieved above and beyond the incidental physical activity one might accumulate in the course of daily living. Adults are encouraged to participate routinely in exercise<br>modalities and contexts that are sustainable, enjoyable, safe and reasonably achievable.	Two "purposes" above must be achieved through additional physical activities which are beyond normal daily ones.<br>Adults are encouraged to participate in exercise that are continuous, fun, safe and adequate on a regular basis	• Should and must is used interchangeably in Korean.
Paragraph 3	These guidelines are appropriate for adults (aged 18-64)<br>with chronic spinal cord injury (at least one year post-onset, neurological level of injury C3 and below), from traumatic or non-traumatic causes, including tetraplegia and paraplegia, irrespective of sex, race, ethnicity or socio-economic status.	Regardless of gender, race, or socioeconomic level, this exercise guideline is recommended to adults with chronic quadriplegic or paraplegic spinal cord injury due to traumatic or non-traumatic causes, who have been injured for minimum 1 year, have C3 or below injury, and are between ages 18 to 64.	• In a Korean context, "Ethnicity" makes the text a bit weird since it is not multi-cultural nation. (there are many foreigners living in Korea but somehow, we do not consider/use the term ethnicity much.<br>• To make the context smoother and natural, some phrases were into one phrase or were changed in an order.
Paragraph 4	Before starting an exercise programme, adults with SCI should consult with a	Consult **a medical specialist** who knows well about the types and amount of exercises that are adequate	• Before starting an exercise programme, adults with SCI should consult with a health professional who is

	health professional who is knowledgeable in the types and amounts of exercise appropriate for people with SCI. Individuals with a cervical or high thoracic injury should be aware of the signs and symptoms of autonomic dysreflexia during exercise.	for adults with spinal cord injury **before starting this exercise program.** If you have a cervical or upper thoracic cord injury, be aware of the symptoms of autonomic hyperreflexia which may occur during the exercise.	knowledgeable in the types and amounts of exercise appropriate for people with SCI. – The order of phrases are changed to make it natural<br>• Individuals with a cervical or high thoracic injury should be aware of the signs and symptoms of autonomic dysreflexia during exercise. – 'If phrase' has been used to make it natural in a Korean language context.
Paragraph 5	For adults who are not already exercising, it is appropriate to start with smaller amounts of exercise and gradually increase duration, frequency, and intensity, as a progression toward meeting the guidelines. Doing exercise below the recommended levels may or may not bring small changes in fitness or cardiometabolic health.	**If you have not started any exercise yet,** start with a light one, then gradually increase duration, frequency, and intensity to meet the guideline. Exercise below recommended level can also improve metabolic fitness of cardiovascular system.	• For adults who are not already exercising, it is appropriate to start with smaller amounts of exercise and gradually increase duration, frequency, and intensity, as a progression toward meeting the guidelines. - The order of phrases are changed to make it natural.<br>• The term "cardiometabolic" has no equivalent word in Korean, and it was translated into 'metabolic fitness of cardiovascular system'.
Paragraph 6	The risks associated with these guidelines are minimal when managed in consultation with a health care professional who is knowledgeable in spinal cord injury.	Consulting **a medical specialist** who knows well about spinal cord injury and managing accordingly will minimize the risk associated with this exercise guideline.	• In the context, 'health care professional' was translated into 'medical specialist'.
Paragraph 7	The guidelines may be appropriate for individuals with a SCI less than 12 months post-onset, aged 65 years or older, or living with comorbid conditions. There is currently insufficient scientific evidence to draw firm conclusions about the risks and benefits of the guidelines for these individuals. These individuals should consult a health care provider prior to beginning an exercise programme.	Although it is also recommendable for those who have been injured for less than 12 months or are over age 65, or have complications, **a medical specialist** must be consulted in such cases before starting the exercise program.<br>There is not enough scientific evidence to confirm the risks and benefits of the guideline for them.	• The order of phrases are changed and the phrases were merged into one sentence to make it natural<br>• In the context, 'health care professional' was translated into 'medical specialist'.
Paragraph 8	Exceeding these exercise guidelines would be expected to yield additional cardiorespiratory fitness and muscle strength and cardiometabolic health	Exercising beyond the recommended guideline may or may not provide additional improvement in cardiopulmonary fitness and muscular strength, as well as metabolic fitness of cardiovascular system,	• When the two sentences of Original Text were translated directly into Korean, it was difficult to understand. So it shows a big difference between two texts.

	benefits. However, there are insufficient data to comment on the risks associated with a person with SCI exceeding these guidelines.	however, not enough evidence exists on the relevant risk of over-exercising.	• 'Data' was changed to 'evidence'
Subtitle Fitness	Fitness<br>For cardiorespiratory fitness and muscle strength benefits, adults with SCI should engage in at least:<br>20 minutes of moderate to vigorous intensity aerobic exercise, 2 times a week<br><br>3 sets of strength-training exercises for each major functioning muscle group, at a moderate to vigorous intensity, 2 times a week	The improvement of cardiorespiratory fitness and muscle strength benefits<br><br>Participate in the following exercises more than twice a week to improve cardiopulmonary fitness and muscular strength.<br><br>Minimum 20 minutes of **mid- to high-intensity** aerobic exercise, 2 times a week<br><br>Minimum 3 sets of **mid- to high-intensity** global muscle exercise of upper or lower limbs for over 20 minutes, 2 times a week	• 'moderate to vigorous intensity' in the original text became 'mid- to high-intensity' in the back-translation.<br>• 'major functioning muscle group' was indirectly translated into 'major muscle of upper or lower limbs(extremities)'
Subtitle Cardiometabolic health	Cardiometabolic health<br>For cardiometabolic health benefits, adults with SCI are suggested to engage in at least:<br>30 minutes of moderate to vigorous intensity aerobic exercise, 3 times a week	The improvement of metabolic fitness of cardiovascular system<br>Participate in the following exercises more than three times a week to improve metabolic fitness of cardiovascular system.<br><br>Minimum over 30 minutes of mid- to high-intensity aerobic exercise, **3** times a week.	• 'at least: 30 minutes of moderate to vigorous intensity' was translated 'minimum over 30 minutes of mid- to high-intensity'.

Thailand

	Original Text	Back Translation	Comments
Title	Scientific exercise **guidelines** for adults with spinal cord **injury**	Scientific based exercise **guideline** for adults with spinal cord **injuries**	• In Thai there were no single or plural form for showing pronoun, so it could make a difference in back translation but it still have the same meaning.
Paragraph 1	These exercise **guidelines** provide minimum thresholds for achieving the	This exercise **guideline** provides minimal limit of exercise for these benefits	• In Thai there were no single or plural form for showing pronoun, so it could make a difference in back

	following benefits:<br>• **improved** cardio**respiratory** fitness and muscle strength<br>• improved cardiometabolic health	To **increase** cardio**pulmonary** fitness and increase muscle strength<br>To increase cardiometabolic capacity	translation but it still have the same meaning.<br>• The word improved and increase have the same meaning in Thai. And we use the same verb for all tense (past, present, future tense) in Thai<br>The word respiratory and pulmonary have the same meaning in Thai.
Paragraph 2	The guidelines should be achieved above and beyond the incidental physical activity one might accumulate in the course of daily living. Adults are encouraged to participate routinely in exercise<br>modalities and contexts that are sustainable, **enjoyable**, safe and reasonably achievable.	This guideline recommends patients to have more physical activities than just activities for daily living. Adults should be encouraged to exercise regularly with sustainable, **satisfactory**, safe and practical contexts.	• The word enjoyable is a little bit more positive meaning compared with satisfactory in Thai. So we change the word in Thai to the another word which give meaning more similar to enjoyable after second review.
Paragraph 3	These guidelines are appropriate for adults (aged 18-64)<br>with chronic spinal cord injury (at least one year post-onset, neurological level of injury C3 and below), from traumatic or non-traumatic causes, including tetraplegia and paraplegia, irrespective of sex, race, ethnicity or socio-economic status.	This guideline is applicable for adults (age 18- 64 years old) with chronic spinal cord injuries (more than 1 year and having injury at C3 level or below) from traumatic and non-traumatic causes including paraplegia and quadriplegia regardless of sex, race, ethnicity or socioeconomic status.	
Paragraph 4	Before starting an exercise programme, adults with SCI should consult with a **health professional** who is knowledgeable in the types and amounts of exercise appropriate for people with SCI.<br>Individuals with a cervical or high thoracic injury should be aware of the signs and symptoms of autonomic dysreflexia during exercise.	Before starting the exercise program, spinal cord injury patients should consult with **medical personnel** who has knowledge about types and suitable amount of exercise for spinal cord injury patients. Patients who have cervical and upper thoracic level of spinal cord injuries should be aware of signs and symptoms of autonomic dysreflexia during exercise.	• The word health professional and medical personnel have the same meaning in Thai.
Paragraph 5	For adults who are not already exercising, it is appropriate to start with smaller amounts of exercise and gradually increase duration, frequency, and intensity, as a progression	For those who have never done any exercise before should start with small amount of exercise and gradually increase duration, frequency and intensity of exercise to the recommended dose. Performing	• The word 'may' and 'might' have the same meaning in Thai.<br>• The word 'fitness' and 'physical fitness' have the same meaning in Thai.

	toward meeting the guidelines. Doing exercise below the recommended levels **may** or may not bring small changes in **fitness** or cardiometabolic health.	exercise less than the recommended levels **might** or might not cause little change in **physical** and cardiometabolic fitness.	
Paragraph 6	The risks associated with these guidelines are minimal when managed in consultation with a **health care professional** who is knowledgeable in spinal cord injury.	Relevant risk occurred by following this guideline is very low when it is done by **medical personnel** who has knowledge about exercise for spinal cord injury patients.	• The word health care professional and medical personnel have the same meaning in Thai.
Paragraph 7	The guidelines **may** be appropriate for individuals with a SCI less than 12 months post-onset, aged 65 years or older, or living with comorbid conditions. There is currently insufficient scientific evidence to draw firm conclusions about the risks and benefits of the guidelines for these individuals. These individuals should consult a **health care provider** prior to beginning an exercise programme.	This guideline **might** be applicable to patients who suffered from spinal cord injuries for less than 12 months, aged more than 65 years old, or having other comorbid conditions. Currently, there is still not enough scientific evidence to conclude that this guideline will have risks or benefits for these patients. These patients should consult with **medical personnel** before starting exercise program.	• The word 'may' and 'might' have the same meaning in Thai.<br>• The word health care provider and medical personnel have the same meaning in Thai.
Paragraph 8	Exceeding these exercise guidelines would be expected to yield additional cardiorespiratory fitness and muscle strength and cardiometabolic health benefits. However, there are insufficient data to comment on the risks associated with a person with SCI exceeding these guidelines.	Performing exercise exceeding the recommendations might increase more cardiopulmonary fitness, muscle strength and cardiometabolic capacity than expected. However, there is no sufficient data to indicate possible risks.	
Subtitle Fitness	Fitness<br>For cardiorespiratory fitness and muscle strength benefits, adults with SCI should engage in at least:<br><br>20 minutes of moderate to **vigorous** intensity aerobic exercise, 2 times a week<br><br>3 sets of strength-training exercises for each	Physical fitness<br>To increase cardiopulmonary fitness and muscle strength in adults with spinal cord injuries, patients should exercise at the minimum of the followings:<br><br>Moderate to **high** intensity aerobic exercise for 20 minutes twice a week	• The word vigorous and high have the same meaning in Thai.<br>• The word major and main have the same meaning in Thai.<br>• And the way we arrange sentence in Thai is difference from English to make it easier to understand.

	**major** functioning muscle group, at a moderate to vigorous intensity, 2 times a week	Moderate to high intensity strengthening exercise of each **main** muscle group for 3 sets twice a week	
Subtitle<br>Cardiometabolic health	Cardiometabolic health<br>For cardiometabolic health benefits, adults with SCI are suggested to engage in at least:<br>30 minutes of moderate to vigorous intensity aerobic exercise, 3 times a week	Cardiometabolic capacity<br>To increase cardiometabolic capacity in adults with spinal cord injuries, patients should exercise at the minimum of the followings:<br><br>Moderate to high intensity aerobic exercise for 30 minutes three times a week	

- **Any difficulties you found in translating the Guidelines (if there is no comment, please leave it blank)**

Indonesia

English and Indonesian language has different grammar. Sometimes, an English vocabulary can have some meaning in Indonesian language. So when we translate a sentence from English to Indonesian language and then translate it back to English, some words can be different. It can still have the same meaning, but it can also make different interpretation. For examples: *high thoracic injury* become *upper back injury*, *neurological level of injury C3* become *level of injury on cervical 3*.

Japan

・In English original text, the phrase that is 'irrespective of sex, race, ethnicity or socio-economic status' comes at the end of the sentence, however, Japan put it in the beginning. Interestingly, the back translation of Japan put it back to the end of the sentence.

・we avoided translating word for word or the meaning can be weird in a Japanese context.

Korea

As there were no equivalent words of cardiometabolic health, major functioning muscle group, and health care professional, we try to find relevant words in the context.

In English original text, the phrase that is 'irrespective of sex, race, ethnicity or socio-economic status' comes at the end of the sentence, however, Japan put it in the beginning.

Interestingly, the back translation of Japan put it back to the end of the sentence.

Thailand

English and Thai language has different grammar such as no single or plural form in Thai and same verb for all tense (past, present, future tense) in Thai. Sometimes, English words can have same meaning when translate into Thai such as “improve and increase”, “respiratory and pulmonary”, “health (care) professional, health care provider and medical personnel” and “may and might”. So when we forward translate a sentence from English to Thai language and then translate it back to English, some words can be different. However, it can still have the same meaning.

- **What kind of questions did reviewers have? How many people had questions about ‘how’ [guidelines tell us what to do, not how to do it] and if any, what did they ask?** (These questions tell us if supporting resources are needed to support clinical practices of the guidelines)

Indonesia

The questions of two people who asked about 'how' were:

1. If the patients with 0 muscle power (MMT result are 0), especially for tetraplegia patients, what kind of exercise or how they do the exercise?
2. For the exercise prescription, should we add time for the warm up and cooling down? (the guidelines only say 20 minutes or 30 minutes moderate to vigorous aerobic exercise)

Japan

As the guidelines were a bit unclear, the patents who participated in the reviews asked where and how to exercise

Korea

The patients asked how hard moderate and vigorous intensity should be.
Through the reviews there was active argument on ‘major functioning muscle group’

Thailand

The patient asked about what is the difference between cardiorespiratory fitness and cardiometabolic health? (there is only medical term in Thai for these words but no general term for patient)

The patient asked about meaning of

1. 3 sets (how many times of exercise for 1 set?)
2. major functioning muscle group (which muscle is major functioning muscle group?)

- **The processes of applying terminologies (Please add any terms or expressions if necessary)**

		**Neurological level of injury C3**	**Cervical**	**High thoracic injury**	**Autonomic dysreflexia**	**Tetraplegia**	**Paraplegia**	**Major functioning muscle group**
**Indonesia**	**Translation**	level cedera saraf leher nomor 3	leher	tulang punggung atas	disrefleksia otonomik	kelemahan anggota gerak atas dan bawah	kelemahan kedua tungkai bawah	kelompok otot bahu dan pinggul
	**1st Review**	level of injury on cervical 3	cervical	upper back trauma	autonomic dysreflexia	weakness of upper and lower extremity	weakness of lower extremity	shoulder and hip muscles
	**2nd Review**	level of injury on cervical 3	cervical	upper back injury	autonomic dysreflexia	weakness of four limbs	weakness of both lower limbs	shoulder and hip muscles
**Japan**	**Translation**	神経学的損傷レベル C3 以下	頸部	高位胸髄損傷	自律神経失調症	四肢麻痺	対麻痺	主要な運動を担う筋群
	**1st Review**	Neurological level of injury C3	Cervical	High thoracic injury	autonomic dysreflexia	Tetraplegia	Paraplegia	Each big muscle

	**2nd Review**	Neurological level of injury C3	cervical	High thoracic injury	autonomic dysreflexia	tetraplegia	Paraplegia	Major functioning muscle group
	**3rd Review**	Neurological level of injury C3	cervical	High thoracic injury	autonomic dysreflexia	tetraplegia	Paraplegia	Major functioning muscle group
**Korea**	**Translation**	경수 3 번의 신경학적 수준	경수	상부 흉수손상	자율신경과반사증	사지마비	하지마비	상지 혹은 하지의 큰 근육
	**1st Review**	Neurological level of injury C3	cervical	High thoracic injury	autonomic dysreflexia	tetraplegia	Paraplegia	Major muscles of upper and lower limbs(extremities)
	**2nd Review**	Neurological level of injury C3	cervical	High thoracic injury	autonomic dysreflexia	tetraplegia	Paraplegia	Major muscles of upper and lower limbs(extremities)
**Thailand**	Translation	ไขสันหลังบาดเจ็บที่คอระดับที่ 3	คอ	บาดเจ็บระดับทรวงอกส่วนบน	ภาวะระบบประสาทออโตโนมิกผิดปกติ	อัมพาตแขนขาสองข้าง	อัมพาตท่อนล่าง	กลุ่มกล้ามเนื้อทำงานหลัก
	1st Review	Neurological level of injury C3	cervical	High thoracic injury	autonomic dysreflexia	Tetraplegia	Paraplegia	Major functioning muscle group
	2nd Review	Neurological level of injury C3	cervical	High thoracic injury	autonomic dysreflexia	Tetraplegia	Paraplegia	Major functioning muscle group

		**Cardiometabolic health**	**Medical care professional**	
**Japan**	**Translation**	心血管と代謝の改善		
	**1st Review**	Cardiovascular and metabolic improvement		
	**2nd Review**	Cardiovascular and metabolic improvement		
**Korea**	**Translation**	심혈관계대사 능력	의료전문가	
	**1st Review**	Cadiovascular metabolic capacity	medical professional	
	**2nd Review**	Cadiovascular metabolic capacity	medical professional	
Thailand	Translation	สมรรถภาพของระบบหัวใจและเมตาบอลิก	บุคลากรทางการแพทย์	
	1st Review	cardiometabolic capacity	medical personnel	
	2nd Review	cardiometabolic capacity	medical personnel	
