# Supplementary figures and images for: Cultural validation and language translation of the scientific SCI exercise guidelines for use in Indonesia, Japan, Korea, and Thailand
Source: J Spinal Cord Med. 2021 Jul 6;45(6):821–32. doi: 10.1080/10790268.2021.1945857 (PMC9661985; doi:10.1080/10790268.2021.1945857)

### Supplementary File 3 PRISMA Flow Diagrams

#### English

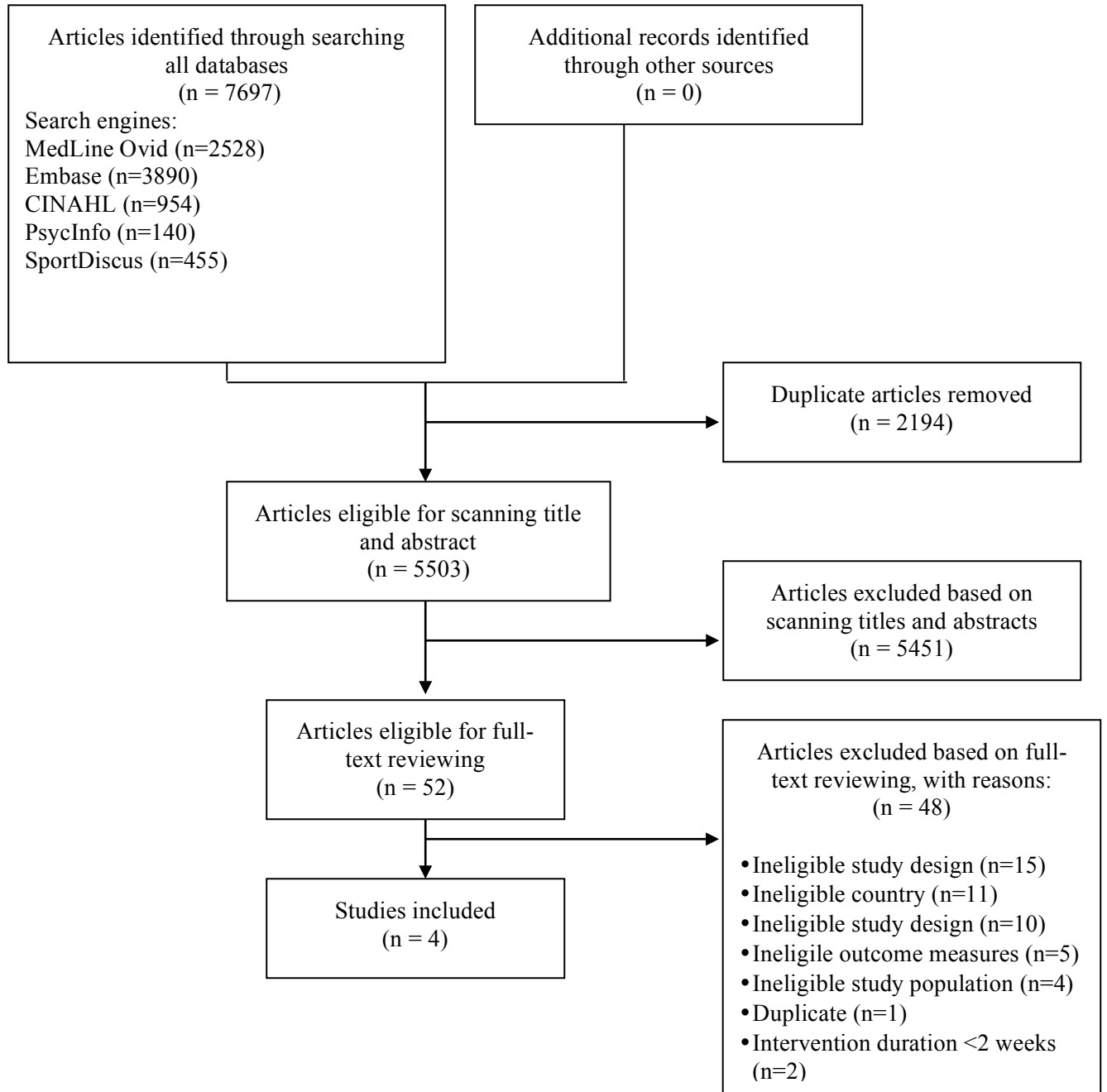

## Indonesia

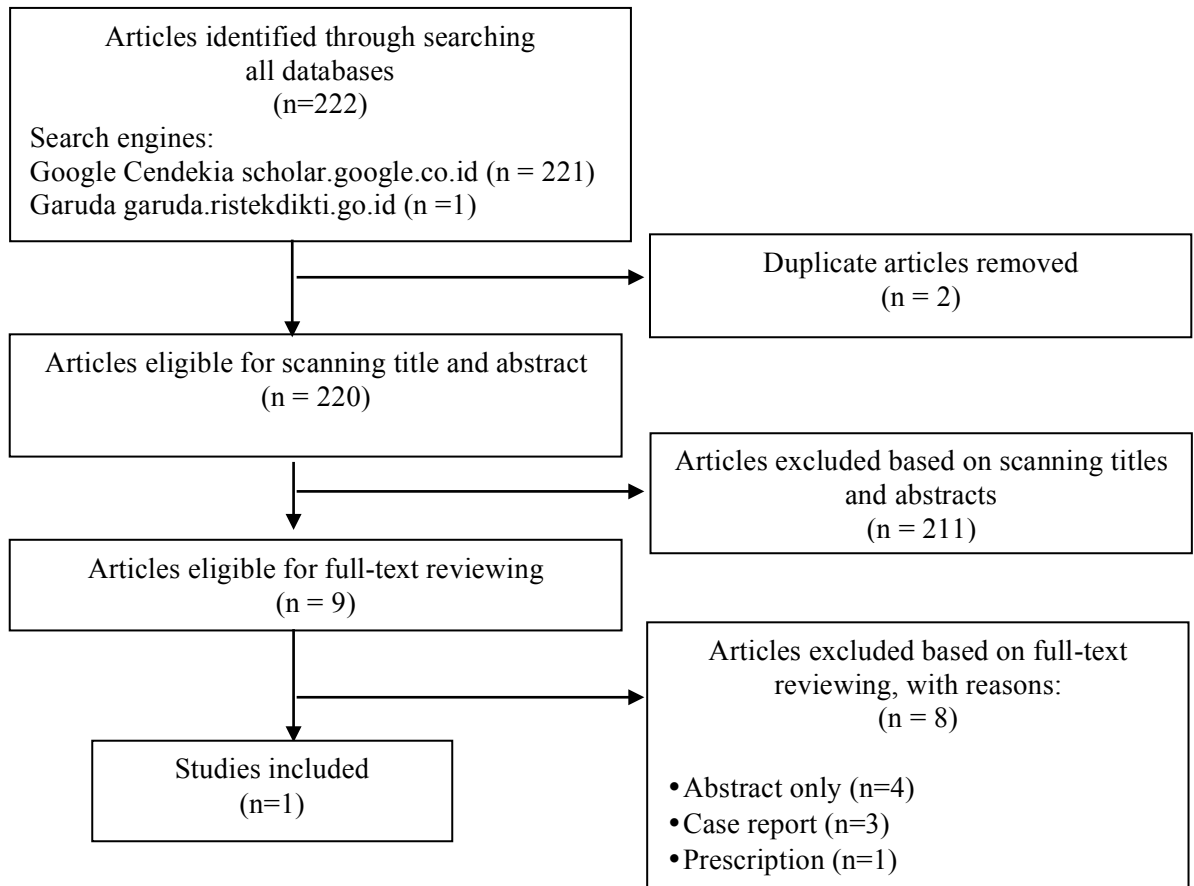

## Japan

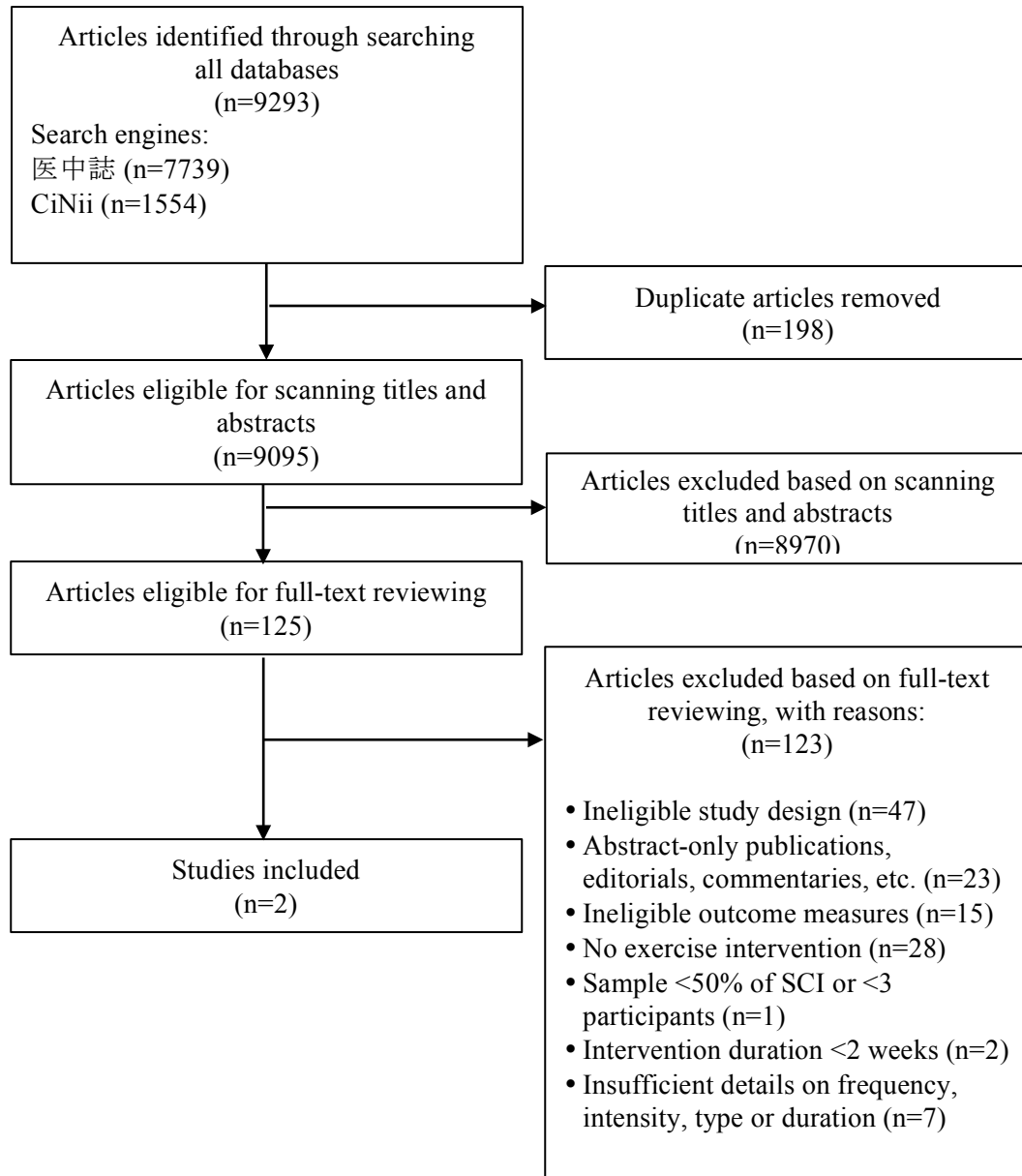

## Korea

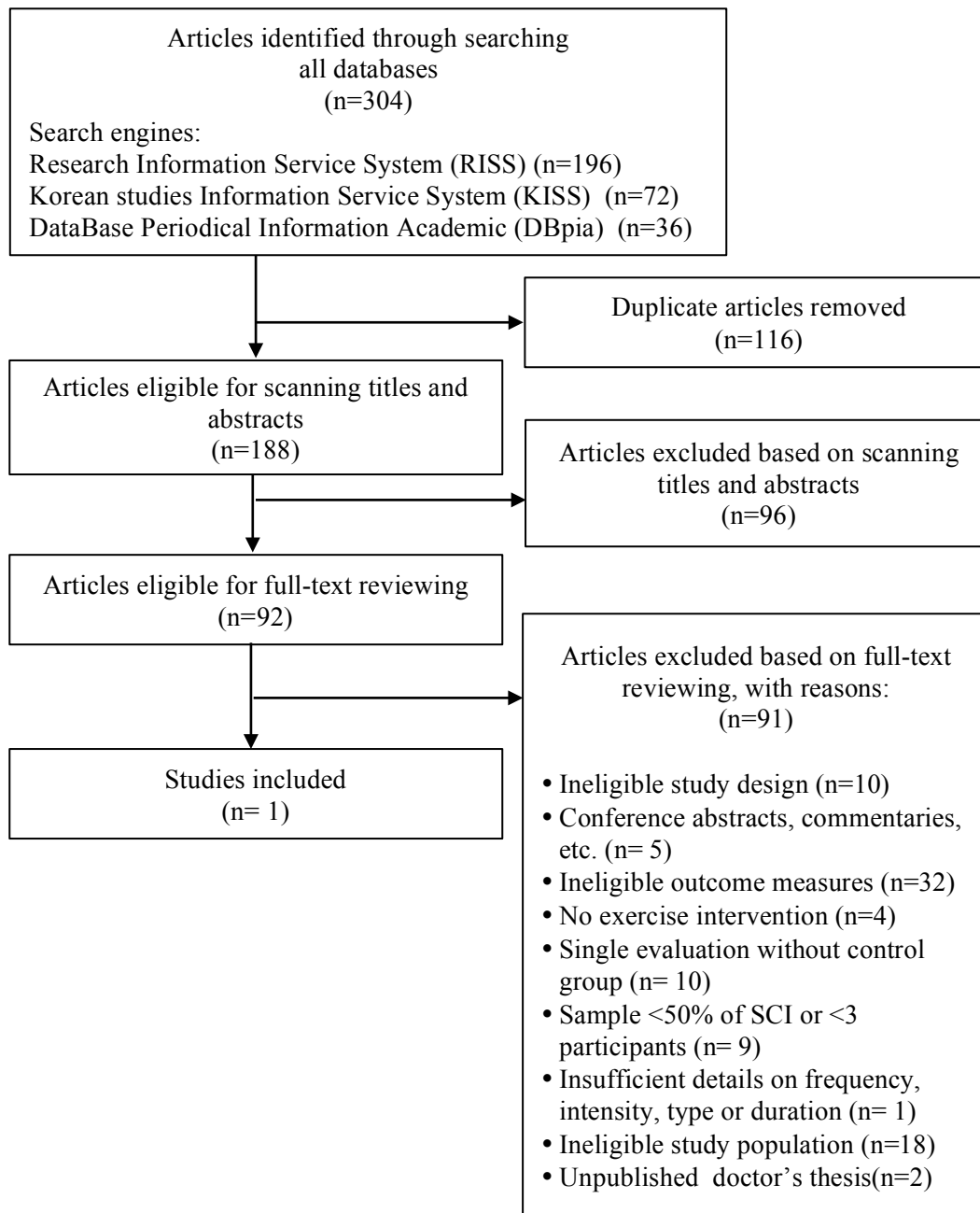

## Thailand

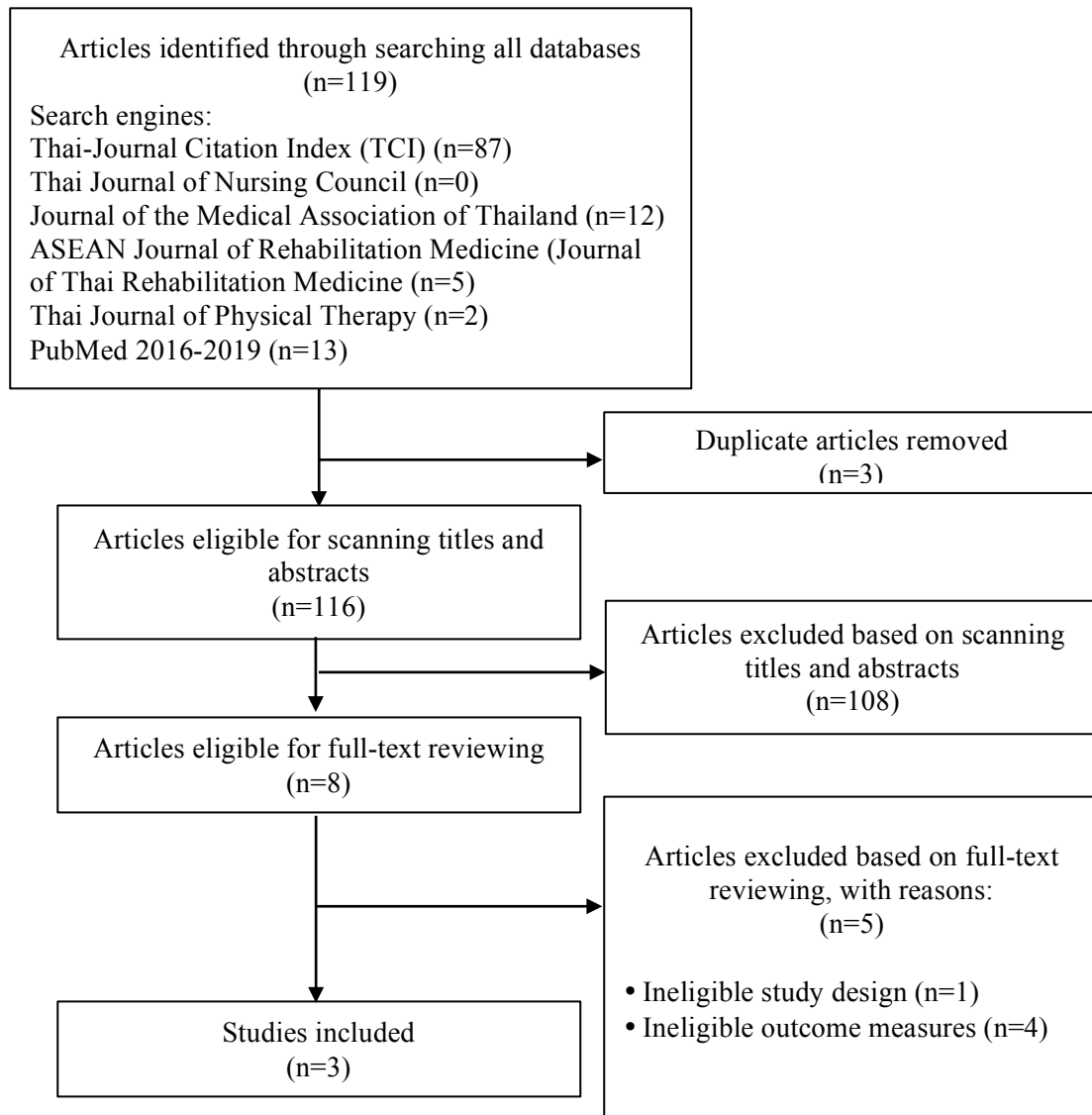

Supplement: Supplemental Material [file YSCM_A_1945857_SM8537.zip › Supplementary File 3 PRISMA Flow Diagrams.pdf]
